# From inner to dyadic connection: the role of mindfulness in mother–infant interaction during the first year of life

**DOI:** 10.3389/fnbeh.2024.1398042

**Published:** 2024-08-08

**Authors:** Ilenia Passaquindici, Massimiliano Pastore, Odette Nardozza, Francesca Lionetti, Giulio D’Urso, Riccardo Palumbo, Mirco Fasolo, Maria Spinelli

**Affiliations:** ^1^Department of Neuroscience, Imaging and Clinical Sciences, University G. d’Annunzio Chieti-Pescara, Chieti, Italy; ^2^Department of Developmental Psychology and Socialization, University of Padua, Padua, Italy; ^3^Department of Psychology, University G. d’Annunzio Chieti-Pescara, Chieti, Italy

**Keywords:** mindfulness, mindfulness-based interventions, physiological attunement, mother–infant interaction, parenting stress

## Abstract

**Introduction:**

Mother–infant attunement is fundamental to supporting infant socio-emotional development. Based on the assumption that we connect better with others if we are aware of and connected with our own experience, mindfulness could affect the maternal ability to attune to the infant. However, little is known about this topic in the first year of life. Study 1 aimed to investigate the role of maternal dispositional mindfulness and mindful parenting in mother–infant physiological and behavioral attunement at 3 months of age. Study 2 aimed to explore the effect of a mindfulness-based intervention not specific to parenting experience on mother–infant behavioral and physiological attunement and on maternal wellbeing at 9 months of age.

**Methods:**

In Study 1, mother–infant (*n* = 67) behavioral and physiological attunement (i.e., co-regulation and RSA) were collected simultaneously each 20 s during face-to-face interaction. Mothers completed questionnaires about their dispositional mindfulness and mindful parenting. In Study 2, mother–infant dyads were randomly divided into a control (*n* = 20) and an intervention group (*n* = 29). The intervention group attended a 5-week mindfulness-based intervention. At T1 and T2, the same procedure described in Study 1 was applied and mothers reported about their wellbeing.

**Results:**

Results showed that maternal mindfulness was associated with high physiological and behavioral attunement at 3 months and with more positive maternal behaviors and less stress at 9 months. Analysis evidenced a slight improvement in the intervention group in maternal dispositional mindfulness and a reduction in parenting stress at T2.

**Discussion:**

Findings from both studies suggested that maternal mindfulness could represent a protective factor that could support mothers in fostering better dyadic interactions with their infants. The implementation of mindfulness-based interventions for mothers could have preventive and clinical implications.

## 1 Introduction

Mindfulness—defined as “the awareness that emerges through paying attention on purpose, in the present moment, and non-judgmentally to the unfolding of experience moment by moment” (Kabat-Zinn, 2003, p. 145)—has been widely studied as an intrapersonal phenomenon, indicating individual ability to connect with and regulate personal inner experience (Siegel, 2007). Indeed, mindfulness is generally associated with psychological wellbeing (Bränström et al., 2011; Harrington et al., 2014; Lundwall et al., 2019), good emotion regulation (Roemer et al., 2015; Guendelman et al., 2017; Iani et al., 2019), and low anxiety, depression, and stress (Medvedev et al., 2018). The beneficial effect of mindfulness has been demonstrated even from a physiological perspective. Previous research has shown the potential role of mindfulness in changing adults’ physiological functioning, i.e., reducing physiological stress and improving physiological regulation (see Grecucci et al., 2015; Skoranski et al., 2019 for reviews).

Mindfulness also affects interpersonal functioning and the quality of intimate interactions. Adults’ mindfulness is positively associated with marital satisfaction (Williams and Cano, 2014; Khaddouma et al., 2015), friendship quality (Pratscher et al., 2018), and dyadic synchrony expressed by both the synchronization of behaviors (Haas and Langer, 2014) and physiological arousal (Laurent et al., 2017) with the partner during dyadic interactions.

More recently, the literature has drawn attention to the role of mindfulness in the critical intimate interpersonal relationship between the mother and the child. Building on the premise that maternal mindfulness may enhance not only the maternal connection with her own emotional experiences but also with those of the infant, the limited studies investigating this topic have reported intriguing positive associations between maternal mindfulness and the quality of mother–infant interaction across various developmental stages (e.g., Coatsworth et al., 2010, 2018; Medeiros et al., 2016; Pickard et al., 2018; Potharst et al., 2021; Ben Uriel-Maoz et al., 2023). This led to the development of several mindful-based interventions directed to parents in order to improve their interaction with offspring (e.g., Fernandes et al., 2022; Anand et al., 2023). However, literature on this subject remains limited, with numerous gaps yet to be addressed. Results have shown inconsistency, and only a few studies, primarily relying on self-reports, have examined this topic during infancy, a critical period when infants develop the foundation of socio-emotional competencies primarily through the quality of mother–infant interaction. Understanding whether mindfulness could be deemed a potential protective factor is crucial. Moreover, most studies focused on only one member of the dyad (i.e., the mother or the infant) (e.g., Noroña-Zhou et al., 2022), and none considered both behavioral and physiological dyadic attunement.

### 1.1 The role of maternal mindfulness on mother–infant attunement

The importance of early mother–infant interactions in the first year of life has been well-established, pointing out the short- and long-term positive effects on several child’s developmental domains, such as emotional, social, and cognitive skills (e.g., Feldman and Eidelman, 2005; Feldman, 2012; Cooke et al., 2019; Rocha et al., 2020; Spinelli et al., 2022; Lionetti et al., 2023; Shah et al., 2023).

During infancy, mother–infant interactions are often described as mutually regulated and attuned. Attunement is a dyadic construct that refers to the dynamic and reciprocal adaptation of each partner to the internal and external states of the other interactive partner (Bornstein, 2022). Mother–infant dyadic attunement occurs at different levels of experience, involving not only their coordinated and contingent behaviors (i.e., emotional expressions, movements, and vocalizations) but also a coordinated and shared physiological functioning (i.e., breath, heart rate) (Feldman, 2006, 2007; Spinelli and Mesman, 2018). Given that infants’ communicative skills are still immature during the first months of life, mother–infant attunement is largely associated with the mother’s ability to attune, regulate, and respond appropriately to the infant’s signals (Stern et al., 1985). This ability could be influenced by many maternal individual factors, and mindfulness could be one of those.

When mothers are aware and connected with their own inner experience they might be more able to understand and attune to the infant’s needs, emotions, and communicative cues (Parent et al., 2016; Pickard et al., 2018; Potharst et al., 2021). According to Snyder et al. (2012), mindfulness may foster healthy interactions through a better connection with oneself for two main reasons. The first one is the “quality of presence” that describes the availability to receive whatever the other person brings to the interaction, to sense her own participation in the interaction, and to be aware of her own awareness. By being present the mother is able to perceive and embrace consciously what is happening internally and externally and to connect and attune to the infant’s cues. The second reason is the ability to respond with awareness of what is happening, instead of reacting without awareness. This enables the mindful mother to develop a “space” between perception and action where it is possible to notice her moments of frustration, to regulate her emotions, and to find more functional and adaptive ways to respond to the infant, rather than acting automatically.

This ability to bring non-judgmental, present-centered awareness to the child has been defined as mindful parenting (Pratscher et al., 2019), and its implications for the quality of parent–infant interaction have been examined mainly during childhood and adolescence using parent reports. More mindful parents showed more positive parent–child interactions (Duncan et al., 2015; Medeiros et al., 2016; Zhang et al., 2019), more positive and supportive conflict resolution (Kil and Grusec, 2020; Bird et al., 2021), and shared more positive emotions with their child (Turpyn and Chaplin, 2016) than less mindful parents. During post-partum high maternal mindful parenting facilitated the development of the bond with the infant (Fernandes et al., 2021). Observational studies are limited. Potharst et al. (2021) evidenced an association between some aspects of maternal mindful parenting and the quality of mothers’ behaviors during the interaction with their 0- to 48-month-old infants, such as more gaze to the infant, higher maternal sensitivity, and more positive affect. Furthermore, Laurent et al. (2017) suggested a positive effect of mindful parenting on the mother’s physiological regulation of interactive distress during the still-face procedure, evidenced by steeper cortisol recovery slopes.

Another area of study has explored the role of mindfulness as an individual trait—not specific to parenting experience—known as dispositional mindfulness, in the context of mother–infant interaction, to see whether the ability of the mother to generally focus on her inner experience is related to her attunement with the child. In this sense, maternal dispositional mindfulness assessed during pregnancy was associated with self-reported mother–child bonding during pregnancy (Cohen, 2010; Golmakani et al., 2021) and in the second year of life (Brassel et al., 2020). More mindful pregnant women had better cardiovascular adaptation during pregnancy (i.e., less decrease in cardiac parasympathetic activity) and less emotional distress, both during and after pregnancy, and at 4 months they reported their infants to be more adaptable (Braeken et al., 2017). Maternal dispositional mindfulness can serve as a protective factor in mitigating the adverse effects of depressive symptoms and maternal emotional dysregulation, respectively, on prenatal mother–infant bonding (Hicks et al., 2018) as well as on infant neurobehavioral development (Ostlund et al., 2021). A recent cross-sectional study evidenced how more mindful mothers of 1- to 5-year-old children reported a better capacity to regulate their own emotions during parenting consequently positively affecting their satisfaction, confidence, and pleasure in interacting with their child (Passaquindici et al., 2024).

Similar to what occurs with mindful parenting, very few studies explored the role of maternal dispositional mindfulness during observed mother–infant interaction, yielding mixed findings. Mothers high in dispositional mindfulness exhibited heightened neural perceptual and emotional processing when observing positive moments in video recordings of interactions with their 3-month-old infants, and less when observing negative moments. This suggests an inclination to allocate more attention and perceptual/emotional processing resources to moments of playfulness and joy with their infants as opposed to moments of frustration (Laurent et al., 2018). Maternal dispositional mindfulness during pregnancy, specifically the ability to non-react to inner experience, was associated with maternal-attuned responses to infant distress, but not with maternal sensitivity, at 7–10 weeks post-birth (Pickard et al., 2017) and at 6 months (Pickard et al., 2018).

While these findings suggest a potential relationship between mindful parenting, dispositional mindfulness, and certain maternal and children’s behaviors and physiological functioning, which are relevant indicators of mother–child attunement, the current limitations in the literature prevent definitive conclusions. Given the significance of mother–infant attunement, especially during infancy when the characteristics of dyadic interactions vary notably, further studies are required. Specifically, research investigating these associations during the early years of life, with narrower age ranges, and employing observational methods are necessary.

### 1.2 Mindfulness-based interventions and mother–infant attunement

Based on the positive associations that emerged between mindfulness and the quality of the mother–child relationship and maternal wellbeing, various types of interventions aimed at improving maternal mindfulness have been promoted in both clinical and non-clinical settings. Among those, several modalities were developed including both face-to-face (e.g., Gu et al., 2015; Goldberg et al., 2018) and online (Spijkerman et al., 2016) mindfulness-based interventions.

Interventions focused on mindful parenting have been positively associated with maternal wellbeing when attended during pregnancy and post-partum. Online mindfulness-based interventions, delivered through text messages or audio-recorded formal practices with mindful parenting-specific content, reduced anxiety and stress of pregnant women (Ciciolla et al., 2023; Hulsbosch et al., 2023). Mothers who participated in a face-to-face mindfulness intervention during pregnancy—including mixed contents, e.g., raisin meditation, breathing awareness, body scan, mindful yoga, labor pain cognitive education, and formal and informal meditation homework—reported lower depression and somatic anxiety, and increased mindfulness and life satisfaction (Wang et al., 2023). Face-to-face interventions with practices tailored for mothers—e.g., exercises where mothers learn to focus their mindful attention on the baby and adopt the baby’s perspective—showed benefits for maternal anxiety, stress, and depression when attended during the first year of the infant’s life (Potharst et al., 2017, 2022; Min et al., 2023).

The few studies examining the effects of mindfulness-based interventions during pregnancy on mother–infant interaction have shown positive outcomes. An intervention delivered via text messages, which included instructions on deep breathing, meditation, responding to fetal movements, planning activities, or reading a story to the baby, led to a reported stronger bond with the fetus (Shreffler et al., 2019). Mothers who participated in face-to-face sessions of mindfulness meditation focused on body scan, mindful eating, mindful communication, labor pain management in couples, breastfeeding, and mind/body connection reported better infant emotional regulation at 3 months (Lönnberg et al., 2021).

Observational studies reported mixed results. Zeegers et al. (2019) found an effect of mindful parenting face-to-face training (i.e., meditations in which mothers focus on their child, including joint sessions with the child) in reducing maternal non-attuned mind-mindedness comments and in improving their 0- to 48-month-old children’s responsiveness in vocalization turn-taking, but not on dyadic synchrony and maternal sensitivity. During school age, a face-to-face family-based mindfulness intervention (i.e., audio-recorded meditations based on notice and not judging physiological, emotional, and cognitive reactions in stressful moments of parenting) reduced children’s diurnal and parents’ evening cortisol levels, evidencing a potential benefit on neuroendocrine functioning (Ho et al., 2020).

A recent meta-analysis (Anand et al., 2023) along with a systematic review (Fernandes et al., 2022) has summarized findings that indicate a modestly positive impact of mindful-parenting interventions on the mother–child relationship. However, the authors underlined that the literature is limited to a few studies, with wide variability among participants, and included studies involved mainly mothers of school-aged children. Interestingly, while the reported effect of interventions on maternal wellbeing is medium to high, the meta-analyses did not find evidence of significant improvements in maternal mindful parenting. This suggests that the mechanisms through which some interventions work and others do not do not necessarily relate to mindfulness and need to be further explored.

With the aim of enhancing maternal wellbeing and the quality of mother–child interaction by fostering maternal intrapersonal connection with own moment-to-moment sensations, thoughts, and emotions, interventions also concentrated on improving dispositional mindfulness by engaging mothers in meditative practices or relaxing tasks (Snyder et al., 2012).

Mothers who attended a mindfulness-based intervention delivered on the WeChat app based on pre-recorded videos about the main general mindfulness topic (e.g., mindful breathing and body scan), and on formal and informal homework, have infants with easier temperament at 6 weeks of age (Zhang et al., 2023). An intervention including mindfulness practices not adapted for mothers (e.g., mindful breathing, body, scan, and mindful listening) through a smartphone app reduced symptoms of maternal depression, anxiety, and stress and increased maternal dispositional mindfulness during the post-partum (Bear et al., 2022). The pilot study conducted by Van Gordon and Cowling (2018) demonstrated how mothers of preschoolers who participated in an online 3-day mindfulness-based intervention, composed of a 15-min pre-recorded audio, including body-scan and breathing meditation, exhibited improved mindfulness, and decreased psychological distress and parenting stress.

To the best of our knowledge, only two studies examined the effect of a mindfulness-based intervention, both face to face and during pregnancy, on observed mother–infant interaction with inconsistent findings. Derakhshanpour et al. (2022) evidenced the effects of a mindfulness-based intervention—composed of general mindful practices such as mindful eating, sitting meditations, exercises of visualization, and homework—only on maternal emotional control and not on the mother–infant quality of attachment. Noroña-Zhou et al. (2022), instead, reported evidence of a face-to-face mindfulness-based intervention with both formal and informal exercises—e.g., awareness of breathing, body sensations, thoughts, and feelings—on the sympathetic nervous system activity and regulation after a stressor at 6 months—that are consistent with lower risk for psychopathology and health problems in mothers who participated in the intervention. No significant effects of the intervention were found for infants observed negative behavior and physiological functioning.

In conclusion, although mindfulness interventions for parents appear to be beneficial, the underlying mechanisms remain unclear. Furthermore, there is a lack of observational studies and research on mindfulness-based interventions that foster maternal intrapersonal connection with moment-to-moment sensations, thoughts, and emotions and their impact on mother–infant interaction.

### 1.3 The current study

During infancy, greater infant–mother behavioral and physiological attunement is crucial for supporting the infant’s emotional development (Feldman, 2012). Therefore, understanding whether mindfulness could serve as a protective factor for mother–infant attunement is particularly intriguing.

To address this, we conducted two exploratory observational studies aimed at examining the role of maternal mindfulness on the quality of behavioral and physiological mother–infant attunement during the interaction. Study 1 examined maternal and infant behavioral and physiological functioning during face-to-face interactions at 3 months of age, in relation to maternal dispositional mindfulness and mindful parenting. Study 2 investigated the impact of an online mindfulness-based intervention not specific to parenting experience on mother–infant behavioral and physiological attunement at 9 months of age.

We chose 3 and 9 months that represent two crucial developmental stages with different infant communicative needs (Trevarthen and Hubley, 1978; Trevarthen and Aitken, 2001). Behavioral attunement was assessed using two microanalytical coding systems aimed at evaluating the coordination of the mother’s and infant’s behaviors and affect during interaction. Physiological attunement was measured in both studies by computing the respiratory sinus arrhythmia index (RSA) of both partners. Changes in RSA reflect dynamic adjustments of the autonomic nervous system via the vagus nerve to maintain homeostasis and support growth and restoration (Porges, 2007; Porges and Furman, 2011). Suppression of RSA reflects adaptive regulatory efforts in response to emotionally challenging events, while maintaining or increasing RSA during interpersonal interaction indicates calm and social engagement (Porges, 2007). Simultaneous changes in parent and infant RSA may illuminate the degree to which they are mutually responsive to each other’s physiological functioning during interaction, potentially predicting beneficial effects for the infant (Depasquale, 2020; Miller et al., 2023).

Because the literature on the topic is scarce and not consistent, the nature of these studies was explorative. For Study 1, it was hypothesized that high maternal dispositional mindfulness/mindful parenting would be linked to (1) a strong association between the mother’s and infant’s RSA during interaction and (2) high dyadic symmetrical co-regulation. For Study 2, it was hypothesized that the mindfulness intervention would improve maternal mindfulness, reduce parenting stress, and improve mother–infant behavioral and physiological attunement. These *a priori* expectations were transformed into Bayesian priors as further detailed in the Supplementary material.

## 2 Study 1

### 2.1 Method

#### 2.1.1 Participants

A total of 67 mother–child dyads participated in the study, recruited from the hospitals in urban areas in the center of Italy. All infants were Italian, 49.3% (*n* = 33) were men, and 22.4% (*n* = 15) had one or more siblings. Inclusion criteria were full-term born and healthy infants, with no developmental delays declared at 3 months, according to their family pediatrician. The average maternal age was 35.43 years (SD = 4.58; range = 24–43), and mothers had an average year of 16.52 education (SD = 2.77; range = 8–21), indicating high education level (i.e., bachelor’s degree or higher). All fathers and 83.6% of the mothers (*n* = 56) were employed with a stable job. Parents were informed that the aim of the study was to examine how infants develop the ability to cope with emotional situations, and their written consent was obtained. The families were compensated for their participation with 25 euros. The sample was treated in accordance with the ethical standards outlined by the American Psychological Association and the Italian Association of Academic Psychologists, and the study was approved by the Department Ethics Review Board of the University (name omitted for blind review).

#### 2.1.2 Procedure

At 3 to 4 months of infant’s age (M = 3.30, SD = 0.37), mothers were invited to the laboratory and video-recorded during 3 min of face-to-face free-play interaction (M duration in seconds = 186.91, SD = 10.95). Mothers were instructed to play with their infants as they usually do without objects and were allowed to touch the infant and speak. Before the beginning of the procedure, the dyad was given time to adapt to the laboratory context and the experimental procedure was explained. The room was outfitted with a table and one chair for the mother. Infants were positioned in a highchair on the table in front of the mother and a mirror behind the infant allowed the recording of the behaviors and facial expressions of both partners. The interaction was stopped before the 3 min when the infant was too distressed.

During the interaction, the mother and infant’s physiological activity was recorded applying ECG sensors on the body of both the infant and the mother.

Additionally, mothers completed self-reports to measure maternal mindfulness.

Out of the 67 dyads, full data on dyadic behaviors, physiological functioning, and maternal mindfulness were completed for 45 dyads. No differences between the full sample and the final sample were present on socio-demographic characteristics.

#### 2.1.3 Measures

##### 2.1.3.1 Maternal dispositional mindfulness

Maternal dispositional mindfulness was measured using the Five Facet Mindfulness Questionnaire (FFMQ, Baer et al., 2006). The FFMQ is a 39-item self-report scored on a 5-Likert-type scale from *never or very rarely true to very often or always true* (e.g., “When I do things, my mind wanders off and I’m easily distracted,” “I watch my feelings without getting lost in them,” “I tell myself that I should not be thinking the way I’m thinking”). The questionnaire consists of 5 sub-scales outlining different aspects of mindfulness: (a) observing sensations, perceptions, thoughts, and feelings, (b) describing the experience with words, (c) non-reactivity to inner experience, (d) non-judging of experiences, and (e) acting with awareness. The total scale, used for the present study, consisted of the sum of the mean scores of each scale. Cronbach’s *α* of the total scale was 0.85, similar to the validation study with an *α* for each subscale ranging from 0.75 to 0.91 (Baer et al., 2006).

##### 2.1.3.2 Maternal mindful parenting

Maternal mindful parenting was measured using the Interpersonal Mindfulness in Parenting scale (IMP; Duncan, 2007, 2023). The IMP is a 31-item self-report rated on a 5-point Likert scale from *never true* to *always true*, e.g., “I pay close attention to my baby when we are spending time together,” “I notice how changes in my baby’s mood affect my mood,” “When things I try to do as a parent do not work out, I can accept them and move on.” The questionnaire consists of five scales outlining different aspects of mindful parenting: (a) listening with full attention, (b) emotional awareness of self and child, (c) self-regulation in parenting, (d) non-judgmental acceptance in parenting, and (e) compassion for self and child. The total scale, used in the present study, consisted of the mean scores of all scales. Cronbach’s *α* of the total scale was 0.78, similar to *α* = 0.75 of the validation study (Duncan, 2007).

##### 2.1.3.3 Mother–infant behavioral attunement

Mother–infant observed behaviors were coded with the Revised Relational Coding System (Fogel et al., 2003) to compute dyadic co-regulation. Every dyadic behavior that lasted almost 2 s was coded using Mangold INTERACT Software. Co-regulation can be described along a continuum from mutual adjustment of partners with shared experience via vocal and non-vocal behaviors (i.e., symmetrical) to the absence of orientation of one partner toward the other (i.e., unilateral). We applied the full coding system, but in the present study, we used the durations of symmetrical and unilateral dyadic co-regulation in the analyses. Symmetrical co-regulation included each moment during which both partners adjust their behaviors continuously and mutually based on behaviors of the other and, co-participating to the innovation of the topic with verbal or non-verbal contributions. Unilateral co-regulation was coded when one partner was paying attention or innovating to the other who was engaged in own activities and did not respond or did not pay attention to the partner. Coding was conducted by a trained coder, and an independently trained coder randomly coded 20% of the sessions to calculate inter-observer reliability. The mean *Cohen’s kappa* value was 0.80.

To account for the dynamical changes of dyadic co-regulation across the interaction, the relative durations of symmetrical and unilateral co-regulation every 20 s of the interaction were computed. A minimum of three values per dyad and a maximum of nine were computed (according to the total length of the interaction).

##### 2.1.3.4 Mother–infant physiological functioning

Physiological data of both the mother and the infant were collected with “ENCEPHALAN-EEGR-19/26” (version E_1863_0M manufactured by Medicom MTD Ltd.). The mother’s and infant’s heart rates were recorded simultaneously using disposable electrocardiogram (ECG) electrodes. The negative recording electrode was placed on the mother’s and infant’s right collarbone (i.e., the right clavicle area) and the positive electrode was on the left. A ground electrode was placed on the right deltoid. All data were monitored online, and physiological signals were sampled at 508 Hz. A standard ECG filter of 0.5 to 30 Hz was used for the cardiac signals prior to data storage.

RSA magnitude estimation was obtained using the Porges and Bohrer (1990) technique that includes parsing the component signal into discrete epochs (10–120 s) and then calculating the natural log of the variance in each epoch. In order to explore dynamic changes in physiological regulation of infants and mothers, a critical information for understanding physiological attunement, we extracted a continuous estimate of RSA with a 20 s sliding window applying Abney et al. (2021) implemented MATLAB script.

##### 2.1.3.5 Analytic plan

Analyses were conducted using the statistical software R (R Core Team, 2021), with rstanarm (Goodrich et al., 2020) and brms (Bürkner, 2017, 2018) packages, both integrated with STAN for Markov Chain Monte Carlo (MCMC) sampling (Stan Development Team, 2022). Graphics were created using the ggplot2 package (Wickham, 2016).

First, we explored univariate and bivariate distributions of target variables.

Afterward, we compared and explored a series of regression models adopting a Bayesian approach for estimating parameters and using a mixed-model approach, incorporating the subject random intercept.

The first series of linear regression models explored the additive and interactive effects of infant RSA, IMP total score (mindful parenting), and FFMQ total score (maternal dispositional mindfulness) on maternal RSA. Second, we explored the additive and interactive effects of infant RSA, IMP total score (mindful parenting), and FFMQ total score (maternal dispositional mindfulness) on symmetrical and unilateral co-regulation, applying a series of multivariate models. Models were compared using a series of comparative indexes (see Supplementary materials S1 and S2), and the best model for each dependent variable was retailed and commented. Further details about the analysis are reported in the Supplementary materials S1 and S2.

### 2.2 Results

#### 2.2.1 Descriptives and correlations

Univariate and bivariate descriptives are presented in the Supplementary Table S1 and Supplementary Figure S1. Mother and infant RSA were highly associated. Both mother and infant RSA were positively associated with high maternal dispositional mindfulness (FFMQ total score) and high unilateral co-regulation proportional duration.

#### 2.2.2 Outcome: mother RSA

For mother RSA, we fitted and compared a set of linear models (see Supplementary Table S2 and Supplementary Figures S2, S3). Two models, M09a and M10a (i.e., the model including, respectively, infant RSA × IMP total score and infant RSA × FFMQ total score as predictors), were equivalent and comparatively better than all other tested models, with an evidence ratio (ER) of 0.229/0.219 = 1.046. Comparing these models with their counterparts without interaction (M05a and M06a respectively) allows us to gauge the relative evidence of interaction: ER = 0.229/0.102 = 2.24 for model M09a and ER = 0.219/0.158 = 1.39 for model M10a. This indicates that interactions are not particularly substantial but still interaction models are better than main effect models.

As these two models exhibit similar evidence, we report the results for both.

##### 2.2.2.1 Model 09a: infant RSA × IMP total score

**Figure 1:**
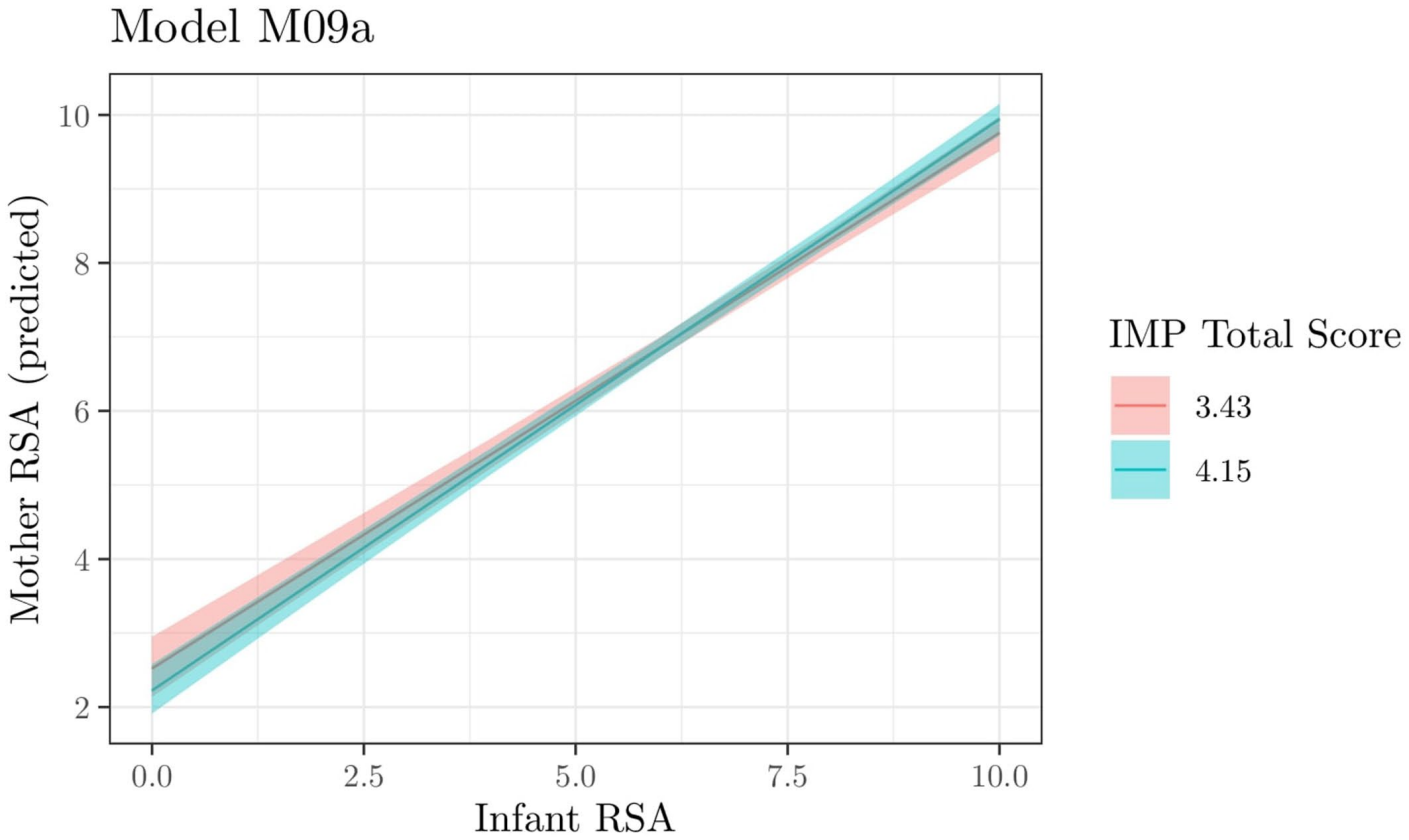
Model M09a. Expected values of the interaction between Infant RSA X IMP Total Score (in colors). The three lines represent values conditioned to the mean and the mean ± the standard deviation of IMP Total Score.

Estimated parameters of posterior distributions for the model are shown in Table 1. The interaction parameter is small, and its 90% CI includes zero among the most likely (*a posteriori*) values. Nevertheless, approximately 88% of the posterior values are positive, indicating a posterior probability of 0.88 for a positive interaction. Figure 1 depicts the expected values of mother RSA as a function of infant RSA (on the *x*-axis) × IMP total score (in colors). The two lines represent values conditioned to the mean (3.79) ± the standard deviation of IMP total score. The RSA of mothers with higher mindful parenting (IMP scores) is strongly associated with infant RSA both when infant RSA is high and when it is low.

**Table 1:** Estimated parameters of posterior distributions for the Models M09a and M10a.

Model M09a	Estimate (SD)	CI 95%
Infant RSA	0.49 (0.23)	0.12–0.86
IMP total score	−0.41 (0.38)	−1.04 to 0.20
Infant RSA × IMP total score	0.07 (0.06)	−0.03 to 00.16

Model M10a	Estimate (SD)	CI 95%
Infant RSA	0.54 (0.17)	0.25–0.82
FFMQ total score	−0.19 (0.34)	−0.75 to 0.37
Infant RSA × FFMQ total score	0.06 (0.05)	−0.02 to 0.14

##### 2.2.2.2 Model M10a: infant RSA × FFMQ total score

**Figure 2:**
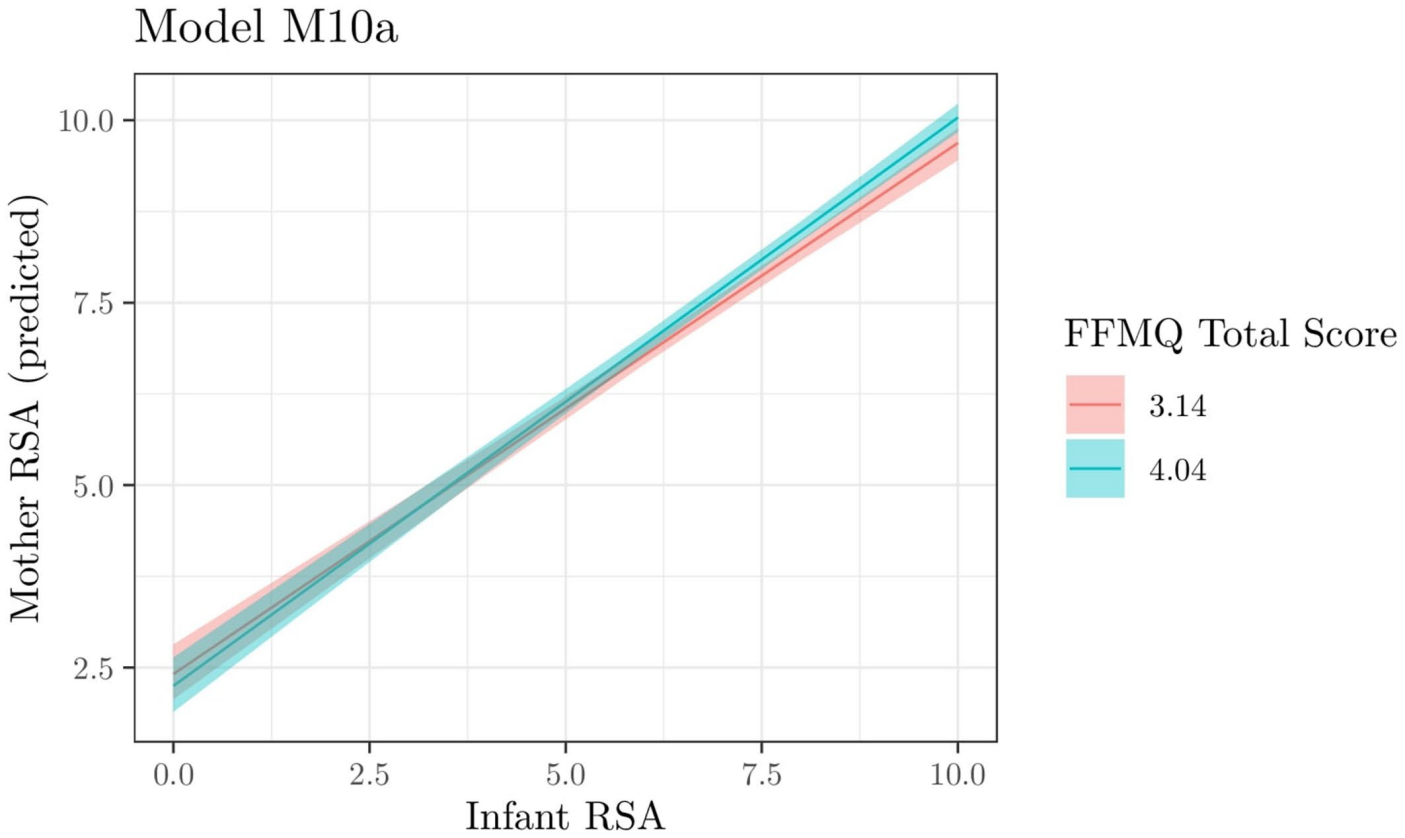
Model M10a. Expected values of the interaction between Infant RSA X FFMQ Total Score (in colors). The three lines represent values conditioned to the mean and the mean ± the standard deviation of FFMQ Total Score.

Estimated parameters of posterior distributions for the model are shown in Table 1. Similar to the interaction parameter of M09a, for this model as well, the interaction parameter is close to 0 and its 90% CI encompasses 0 among the most probable values (*a posteriori*). We observed approximately 89% positive posterior values and a posterior probability of approximately 0.89 indicating that the interaction is positive. Figure 2 depicts the expected values of Infant RSA interaction (on the *x*-axis) × FFMQ total score (in colors). The two lines represent values conditioned to the mean (3.59) ± the standard deviation of the FFMQ total score. The figure is very similar to those of model M09a, indicating that the RSA of mothers with higher dispositional mindfulness (FFMQ scores) is strongly associated with Infant RSA both when infant RSA is high and when it is low.

#### 2.2.3 Outcomes: symmetrical and unilateral co-regulation

For co-regulation variables, we considered a set of multivariate regression models that simultaneously included symmetrical and unilateral co-regulation as dependent variables. Supplementary Table S3 summarizes the model comparison (see Supplementary Figures S4–S7 for diagnostics). The best model (i.e., M08b with IMP total score + time as predictors) has a weight of 0.338 and an ER of 1.5 compared to the second model (i.e., M07b with FFMQ total score + time as predictors). This indicates that the two best models are very close in terms of evidence. Consequently, we reported results for both.

##### 2.2.3.1 Model M08b: IMP total score + time

**Figure 3:**
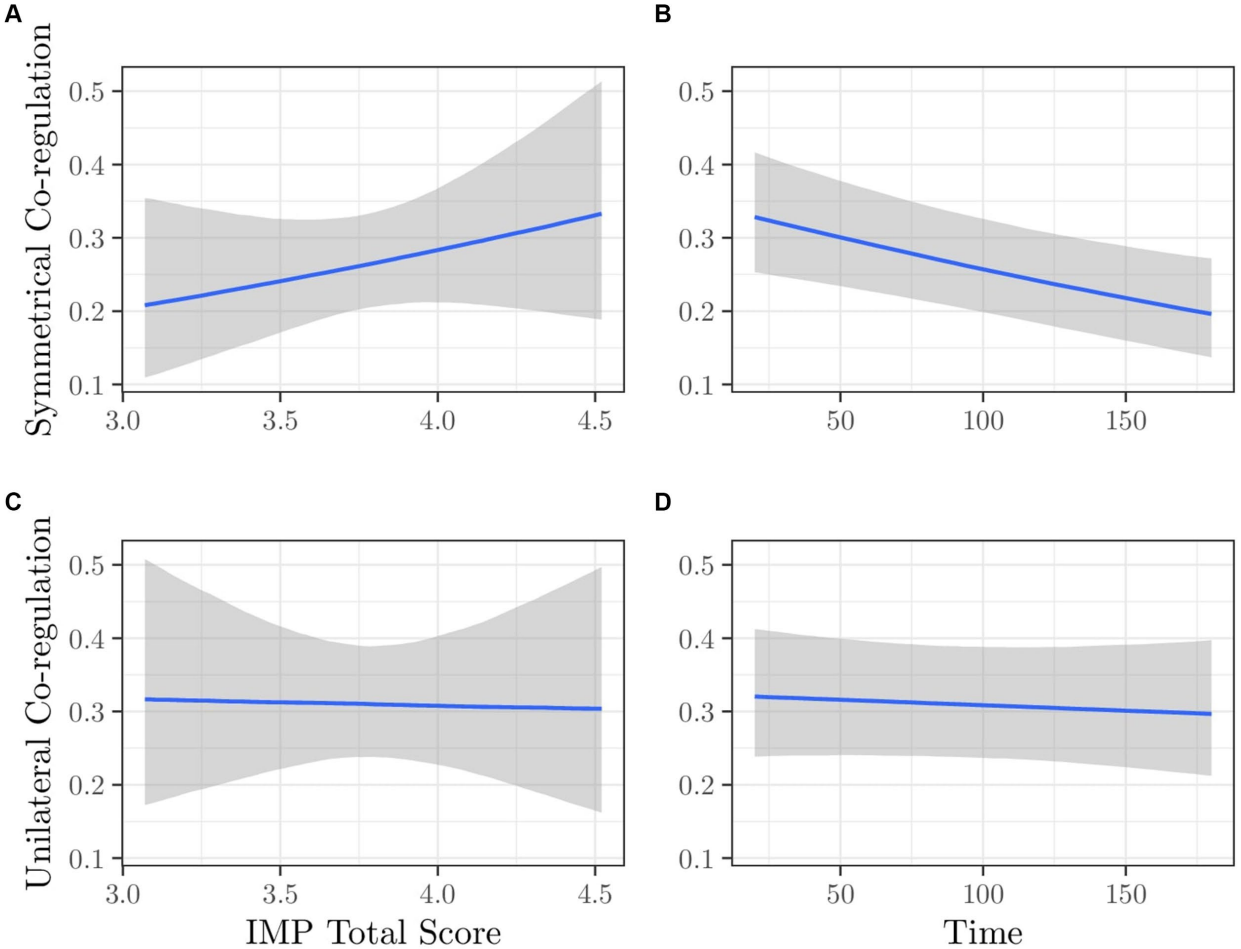
Model M08b. Expected values of Symmetrical Co-regulation as a function of **(A)** IMP Total Score and **(B)** Time; Expected values of Unilateral Co-regulation as a function of **(C)** IMP Total Score and **(D)** Time.

The estimated parameters of posterior distributions for the model for symmetrical co-regulation as the outcome are reported in Table 2. Figure 3 depicts the conditional predictions for symmetrical and unilateral co-regulation as a function of IMP total score and time. Panels (A) and (B) pertain to symmetrical co-regulation, while panels (C) and (D) relate to unilateral co-regulation, respectively. The symmetrical co-regulation increased as a function of IMP total score [panel (A)], indicating that dyads with mothers with high mindful parenting spent more time in symmetrical co-regulation during the interaction. Symmetrical co-regulation also decreased over time [panel (B)]. The unilateral co-regulation did not show any change either as a function of IMP total score [panel (C)] or over time [panel (D)].

**Table 2:** Estimated parameters of posterior distributions for the Models M08b and M07b.

Model M08b	Estimate (SD)	CI 95%
*Symmetrical co-regulation*		
IMP total score	0.44 (0.47)	−0.48 to 1.37
Time	−0.00 (0.00)	−0.01 to 1.00
*Unilateral co-regulation*		
IMP total score	−0.04 (0.50)	−1.03 to 0.95
Time	−0.00 (0.00)	−0.00 to 0.00

Model M07b	Estimate (SD)	CI 95%
*Symmetrical co-regulation*		
FFMQ total score	−0.12 (0.37)	−0.83 to 0.60
Time	−0.00 (0.00)	−0.01 to 0.00
*Unilateral co-regulation*		
FFMQ total score	−0.26 (0.38)	−1.01 to 0.49
Time	−0.00 (0.00)	−0.00 to 0.00

##### 2.2.3.2 Model M07b: FFMQ total score + time

**Figure 4:**
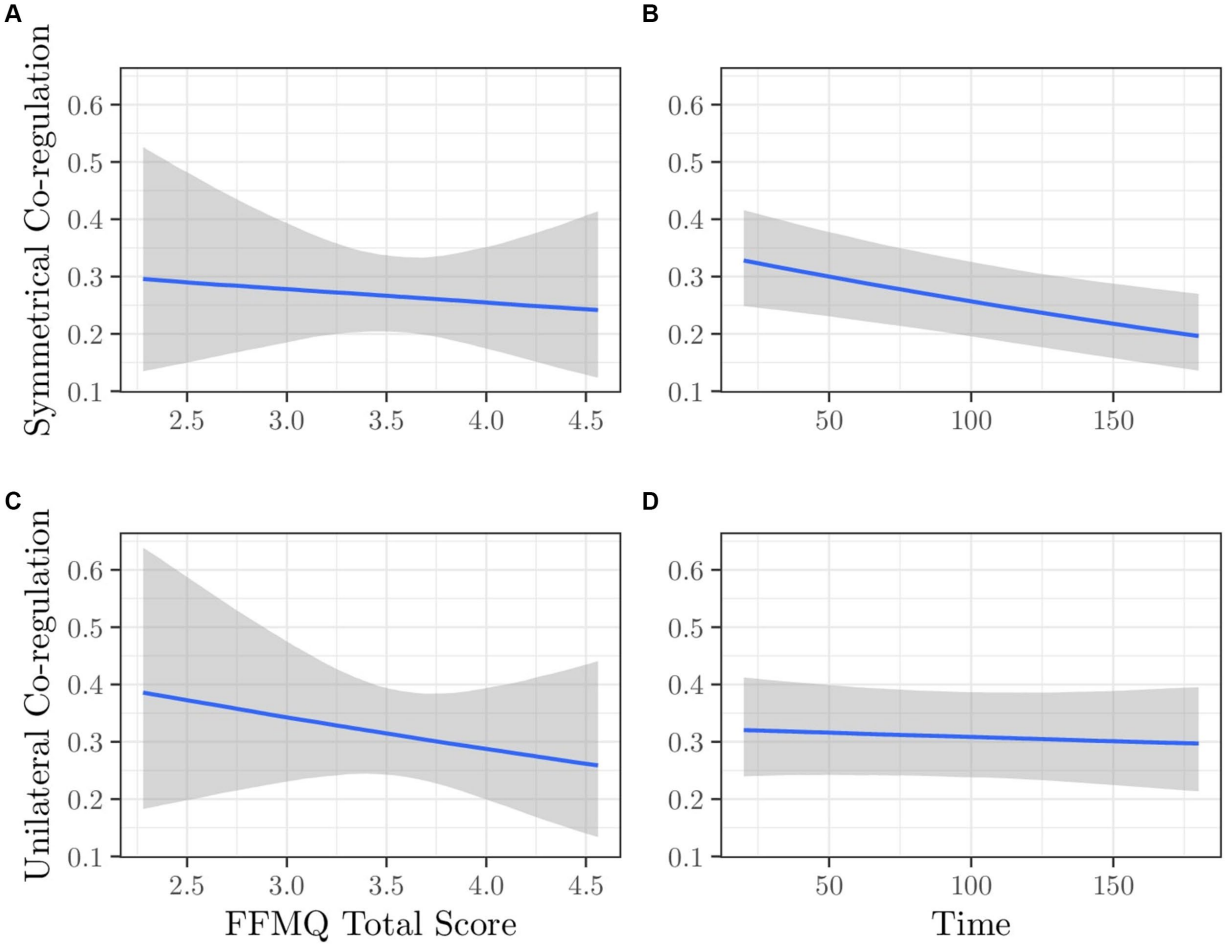
Model M07b. Expected values of Symmetrical Co-regulation as a function of **(A)** FFMQ Total Score and **(B)** Time; Expected values of Unilateral Co-regulation as a function of **(C)** FFMQ Total Score and **(D)** Time.

Estimated parameters of posterior distributions for the model for symmetrical co-regulation and for the model for unilateral co-regulation as outcomes are reported in Table 2. Figure 4 depicts the conditional predictions for symmetrical and unilateral co-regulation as a function of FFMQ total score and time. Panels (A) and (B) pertain to symmetrical co-regulation, while panels (C) and (D) relate to unilateral co-regulation, respectively. In this case, we observed a decrease in symmetrical co-regulation as a function of both FFMQ total score and time. Specifically, dyads with mothers with high dispositional mindfulness spent less time in symmetrical co-regulation during the interaction [panel (A)]. Additionally, symmetrical co-regulation decreased over time [panel (B)], similar to what occurred in model M08b. The unilateral co-regulation did not show any changes over time [panel (D)] but exhibited a decrease as a function of FFMQ total score [panel (C)]. Dyads with mothers with high dispositional mindfulness spent less time in unilateral co-regulation during the interaction.

## 3 Study 2

### 3.1 Methods

#### 3.1.1 Participants

A total of 61 mother–child dyads recruited from social media and in urban areas in the center of Italy participated in this study. All infants, aged between 6 and 13 months (M = 9.14, SD = 1.42), were Italian: 44.3% (*n* = 27) were men; 27.9% (*n* = 17) had one or more siblings. Infants were considered for inclusion if they were healthy, with no developmental delays assessed at pre-test, according to their family pediatrician. The average maternal age was 34.86 years (SD = 4.89; range = 22–46), and mothers had an average of 16.32 years of education (SD = 2.98; range = 8–21), indicating high education level (i.e., bachelor’s degree or higher). All fathers and 80.3% of the mothers (*n* = 49) were employed with a stable job. After parents were informed about the detailed procedure and their right to withdraw at any time, their written consent was obtained. To minimize potential influence on mothers, we informed them that the study aimed to gather their feedback on the mindfulness intervention’s effectiveness in inducing relaxation. The families were compensated for their participation with 25 euros. Of the 61 initial samples, 12 mothers dropped out, mainly from the control group (*n* = 10). The final sample was composed of 49 mother–infant dyads. The sample was treated in accordance with the ethical standards outlined by the American Psychological Association and the Italian Association of Academic Psychologists, and the study was approved by the Department Ethics Review Board of the University.

#### 3.1.2 Procedure

Participants were randomly assigned to the control group (*n* = 20) or to the intervention group (*n* = 29).

At pre-test (T1) and 5 weeks later (T2), the same procedure described in Study 1 was applied for both groups. Dyads were video-recorded for 3 min of free-play interaction (M duration in seconds = 173.37, SD = 25.5), both partners physiological functioning was recorded by applying ECG sensors, and mothers completed mindfulness and parenting stress questionnaires.

Between T1 and T2, the intervention group participated in a 5-week mindfulness-based intervention that started within a few days after the pre-test.

##### 3.1.2.1 Mindfulness intervention

The intervention group participated in a 5-week duration mindfulness-based intervention, developed *ad hoc* for this study by a mindfulness trainer. The intervention consisted of listening to five audios focused on five main mindfulness topics (see Table 3 for details). Mothers received each audio two times on specific days (Monday and Wednesday) of each week of the intervention via WhatsApp, and they were asked to follow the meditation practice. Each audio was 7- to 19-min-long, and it was supplemented by instructions and an explanation of the practice. After the practice, they were asked to fill in a short questionnaire on their enjoyment of the experience to confirm that they completed the practice.

**Table 3:** Details of the 5-week mindfulness-based intervention applied in Study 2.

Session	Practice	Duration in minutes	Description
1	Mindful breathing	6.59	The practice consists of paying attention to the sensation of the breath coming in and out and noticing where you feel it in the body without trying to change it
2	Body scan	19.03	The practice consists of paying attention to body parts and body sensations in a gradual sequence from the feet to the head
3	Mindful walking	10.45	The practice consists of paying attention to our feet and feeling the contact with the floor or ground with each step, with the aim of being aware of our surroundings and body sensations while walking
4	Expanded awareness	19.03	The practice aims to notice that the awareness can be “restricted” and “expanded” whenever desired. From breath to body, from sounds to thoughts, everything can be observed and received with awareness, and each phenomenon arrives, has a peak, an unfolding, and then leaves us
5	Loving kindness	6.54	The practice consists of directing a positive flow of intentions and auspices toward oneself with the consequence of amplifying states of joy and wellbeing

#### 3.1.3 Measures

##### 3.1.3.1 Maternal dispositional mindfulness and maternal mindful parenting

Maternal dispositional mindfulness and maternal mindful parenting were measured, respectively, using the Five Facets Mindfulness Questionnaire (FFMQ; Baer et al., 2006) and the Interpersonal Mindfulness in Parenting scale (IMP; Duncan, 2007, 2023) as in Study 1. Cronbach’s *α* in the current study was 0.77 and 0.81 for the FFMQ total score, and 0.70 and 0.73 for the IMP total score, respectively, at T1 and T2.

##### 3.1.3.2 Behavioral attunement

Infant and mother behaviors were coded using the Infant Caregiver Engagement Phases (ICEP; Weinberg and Tronick, 1999). The ICEP contains a set of mutually exclusive mother and infant codes and combines facial expressions, the direction of gaze, vocalizations, and behavior allowing to code the positive/neutral/negative affect of each member of the dyad (see Tronick et al., 2005 for further details). For the purpose of the present study, we focused on infant positive affect (infant positive), mother social monitor with positive vocalization (mother positive voc.), and mother positive affect (mother positive). Infant positive affect was coded when the infant was smiling at the caregiver and/or cooing, laughing, babbling, or squealing while looking at the caregiver. For mothers, social monitoring with positive vocalization was coded when the mother was focused on the infant’s face with occasional smiles and positive vocalizations (i.e., infant-directed speech, singing, and talking). The social positive engagement was coded if the mother expressed positive affect by smiling, laughing, or making playful faces. The combination of infant and maternal codes was applied to explore behavioral attunement. Specifically, we computed the total duration of the following combinations: mother positive–infant positive (M positive–I positive), mother social monitor with positive vocalization–infant positive (M positive voc.–I positive). The total duration of matched affective states (the infant and the mother sharing the same affect: affective match) and matched attention (the infant and the mother looking at each other, no matter what affect they express: attentive match) were also computed.

The proportional duration of each code on the total duration of the interaction was calculated and used for analyses. As in Study 1, intercoder reliability was assessed by having a second trained observer who independently coded a randomly selected 20% of the videos. Mean *Cohen’s K* across codes was 0.72.

##### 3.1.3.3 Physiological attunement

Mother and infant RSA during the interaction were estimated each 20 s using the same procedure of Study 1. RSA attunement was based on infants’ and mothers’ RSA cross-correlation where the lag-0 cross-correlation was estimated. Each dyad with at least three RSA data (three 20-s intervals) resulted in a measure of physiological attunement (M = 0.54, SD = 0.45, range = −0.998–0.983). Positive correlations indicated a positive association between the mother’s and infant’s RSA; negative correlations indicated a negative association. RSA attunement was available for 37 dyads (19 of the control group and 18 of the intervention group) at T1 and 40 dyads (17 of the control group and 23 of the intervention group) at T2. The reason for the reduction of available data is due mainly to technical reasons (i.e., the ECG sensor moved during the interaction and failed to record data).

##### 3.1.3.4 Parenting stress

Mothers were asked to complete the Parental Distress Scale (PSI PD, Abidin et al., 2006; Guarino et al., 2016) of the Parenting Stress Index—Short Form questionnaire. The questionnaire is commonly used to measure stress in the parent–child system and to identify those caregivers most in need of support and the PSI PD scale indicates the perceived stress related to the parental role. The scale includes 12 items rated from 1 to 5 on a Likert scale (1 = strongly disagree, 5 = strongly agree). High values indicate more parenting stress. Scores were expressed as means. The internal consistency of the current sample was satisfactory and equal to Cronbach’s *α* 0.86 for PSI PD as *α* = 0.80 of the Italian validation study (Guarino et al., 2016).

##### 3.1.3.5 Analytic plan

Analyses were conducted using the same statistical software and packages detailed for Study 1 (see section 2.1.3.5).

First, we explored univariate and bivariate distributions of target variables. Afterward, we compared and explored a series of regression models adopting a Bayesian approach for estimating parameters and using a mixed-model approach, incorporating the subject random intercept for taking into account the pre- and post-intervention assessment.

For exploring the effectiveness of the intervention on outcome variables (mindful parenting, maternal dispositional mindfulness, parenting stress, physiological attunement, symmetrical and unilateral co-regulation), we computed the effect of the interaction between time (T1 and T2) and the intervention condition (control group and intervention group).

### 3.2 Results

#### 3.2.1 Descriptives and correlations at T1

At T1, as expected (see Supplementary Table S4), high correlations evidenced that highly mindful mothers reported experiencing parenting as a less stressful experience.

Concerning individual maternal behaviors, mothers with highly mindful parenting and dispositional mindfulness showed more social monitoring with positive vocalizations.

Maternal mindfulness (mindful parenting and maternal dispositional mindfulness) was associated with high dyadic physiological attunement (measured with RSA cross-correlation), even if the association was low.

Concerning dyadic variables, only the stress perceived in the parenting role (PSI PD) was associated with less mother social monitoring with positive vocalization when the infant was positive and with less affective and attentive match. This indicated that dyads with less stressed mothers showed a better dyadic behavioral attunement.

Univariate statistics of both the intervention and control groups, at both T0 and T1, are presented in the Supplementary Table S5.

#### 3.2.2 Effect of the intervention

##### 3.2.2.1 Outcomes: mindfulness and parenting stress

Supplementary Table S6 displays the interaction parameter (intervention condition × session) estimates with a 90% credibility interval (CI) for mindfulness and parenting stress (IMP total score, FFMQ total score, and PSI PD). See Supplementary Figure S9 for expected values of the models, Supplementary Figure S10 for posterior distributions of interaction parameters (black lines) compared with priors (red lines), and Supplementary Figures S11–S13 for model diagnostics.

Posterior distributions show a trend of increase in the intervention group after the intervention of Maternal Dispositional Mindfulness (FFMQ total score) and a trend of decrease in parenting stress (PSI PD). For both variables, the CIs included zero, but the lower bound of the CI was very low in terms of absolute value and the upper bound of the CI was close to a relatively moderate effect size for FFMQ total score and the opposite situation is present for PSI PD. Almost no changes were identified for mindful parenting (IMP total score). As can we see from the CIs, for IMP total score the interaction value was cantered almost on 0, and the lower CI and upper CI were almost the same in terms of absolute values. Thus, the intervention increased maternal dispositional mindfulness and decreased parenting stress.

##### 3.2.2.2 Outcomes: mother–infant interaction variables

Supplementary Table S7 displays the interaction parameter estimates (intervention condition × Session) for mother–infant interaction variables (see previous section for further details). See Supplementary Figure S14 for expected values of the models, Supplementary Figure S15 for posterior distributions of interaction parameters (black lines) compared with priors (red lines), and Supplementary Figures S16–S22 for model diagnostics.

None of the observed variables showed a trend to increase after the intervention as can be seen by posterior distribution parameters and related CIs.

## 4 General discussion

We conducted two exploratory studies with the main aim of exploring the role of maternal mindfulness in promoting mother–infant interpersonal functioning both physiologically and behaviorally, during spontaneous mother–infant face-to-face interactions at two crucial points of infant development (4 and 9 months). We hypothesized that dyads with high maternal mindfulness would be characterized by better behavioral and physiological attunement (Study 1) and that an intervention aimed at improving maternal mindfulness would improve the quality of dyadic attunement (Study 2).

Overall, the results marginally supported our hypothesis. With respect to Study 1, as expected, maternal mindfulness—dispositional and mindful parenting—was associated with stronger mother–infant physiological attunement during the interaction at 4 months. Even if the strength of the interactions was low, and a high physiological attunement was evidenced in the whole group of dyads, high maternal mindfulness was associated with a stronger association between infant and maternal RSA during the interaction indicating stronger physiological attunement. This association did not emerge in Study 2, with dyads with highly mindful mothers showing only a slight positive association with mother–infant RSA correlations.

In line with previous studies that reported how individual adult mindfulness was associated with physiological attunement during dyadic interactions (e.g., Haas and Langer, 2014; Lampinen et al., 2018), this study expands the emerging literature on the potential role of mindfulness on physiological interpersonal functioning also in mother–infant dyads. Mothers who pay more attention to their own present-moment internal images, thoughts, sensations, and emotions, accepting them without judgment and without getting taken over by them (Kabat-Zinn, 2003) and/or more aware of what happens moment-by-moment during the interaction with their own infants (Pratscher et al., 2019) were more connected with the physiological activation of their infants both when he/she was calm (high RSA), and when he/she was upset (low RSA). This stronger physiological attunement of more mindful mothers to the stressed infant, indicated by high maternal cortisol level, was also previously reported during the stressful condition of the still-face paradigm at 6 months (Laurent et al., 2017).

Because mother–infant interaction supports infant emotional self-regulation, our findings suggest that maternal mindfulness could help mothers to self-regulate their own physiological activation during the interaction in order to serve as an external regulator of their own baby’s affect (Porges, 2001, 2007; Porges and Furman, 2011). However, the high association between mother and infant RSA in our sample (see Study 1) hides the effect of mindfulness. It may be that mindfulness’ effects on physiological attunement are stronger during conditions of stress when the ability of the mother to physiologically regulate her activation is more involved. Future studies should explore this association in other mother–infant conditions to confirm our findings.

The exploration of individual and dyadic behaviors of mothers in association with their mindfulness ability evidenced interesting results at 4 and at 9 months. Individual maternal behaviors were explored only at 9 months, evidencing more time spent talking to the infant with positive vocalizations of more mindful mothers (both high in mindful parenting and in dispositional mindfulness). At 9 months, the infant enters the secondary intersubjectivity phase (Trevarthen and Hubley, 1978; Trevarthen and Aitken, 2001) showing an increasing interest in the exploration of the environment. The role of the mother is to support this exploration by commenting on actions and the focus of interest of the infant. At this stage maternal talking is also crucial to support infant language development. We might suppose that more mindful mothers are more able to adapt their interactive modality to the needs of the infant, spending less time in shared face-to-face positive affect and more in talking and communicating with a more interactive and communicative infant. Longitudinal studies could help understanding whether this interactive modality promotes a more adaptive development in infants of mindful mothers.

Dyadic attunement was explored with different microanalytical coding systems, both focused on the exploration of shared attention and affect. At 4 months, mindful parenting was directly associated with more behavioral attunement, thus more moments of shared attention and affect (i.e., symmetrical co-regulation), whereas maternal dispositional mindfulness was associated with fewer moments during which the mother leads the interaction leaving less space for the infant (i.e., unilateral co-regulation), showing in both cases a better quality of mother–infant co-regulation in dyads with more mindful mothers. Mothers more aware of what is happening here-and-now during the interaction with their infant and more aware of and connected their own here-and-now inner world may be more present and attuned to infant’s behavioral cues, non-reacting to them but responding with awareness, and supporting the baby to co-create a moment of mutual innovation in which each partner is active, anticipates the actions of the other, and adds something new—such as smiles and coos—that lets the interaction to proceed without interruptions (Fogel, 1993). At 9 months, this association was not confirmed, even if a strong association emerged among mindfulness scales and levels of maternal perceived stress experienced during the interaction with their infants. Maybe while mindfulness has an effect on face-to-face dyadic attunement at 4 months, during the second half of the first year of life when the infant increases his/her exploration of the environment, mother–infant attunement should be examined exploring different interactive dimensions. An example could be the analysis of the ability of the mother to follow the focus of interest of the infant such as how much the mother uses verbally appropriate comments.

Because previous studies explored mother–infant interactions including wide infant’s age ranges, comparisons with previous findings are difficult to accomplish. However, our results are consistent with studies reporting that mindful parenting was associated with more positive parent–child interactions, even if at different ages. Specifically, maternal mindful parenting was related to dyadic synchrony of positive facial expressions (Potharst et al., 2021) and more children’s responsiveness in turn-taking (Zeegers et al., 2019) in large age samples including infants, toddlers, and preschoolers up to 48 months. Moreover, mindful parenting was directly related to parental-reported parent–child attachment in preschoolers (Zhang et al., 2019) and to more positive mother–adolescent interactions, more positive parenting, maternal communication skills, and warmth (Duncan et al., 2015).

Altogether, our results suggest a role of mindful parenting and maternal dispositional mindfulness on mother–infant physiological and behavioral attunement, highlighting how mindfulness could promote maternal sensitivity and responsiveness and maternal ability to adapt her behaviors to what previous experiences have taught them about how the infant will respond (Evans and Porter, 2009). However, our findings highlight the need to consider infant age and developmental needs in interpreting findings.

With respect to Study 2, contrary to our hypothesis, dyads of mothers who participated in the mindfulness-based intervention did not improve the quality of mother–infant physiological and behavioral attunement compared to dyads of mothers who did not. However, in dyads of mothers who participated in the intervention there was a trend toward a reduction in parenting stress after participating to the intervention. Consistent with this result, a meta-analysis by Anand et al. (2023) evidenced that the impact of mindfulness-based interventions for parents may be more efficacious intrapersonally (i.e., reduced parental stress), rather than interpersonally, and especially when both parents and infants are engaged in the training. Moreover, a recent systematic review reported that mindfulness-based interventions promoted the quality of mother–child relationship by the promotion of positive parenting attitude toward the child (Fernandes et al., 2022). Considering this, it is possible that the intervention used in our study did not work directly on the mother–infant dyadic attunement but that the effect on dyadic functioning could be mediated by changes in other variables (e.g., maternal wellbeing and parenting styles) that we did not consider due to the limited sample size. Future studies with a larger sample should consider those variables and explore their moderating role on the intervention’s effect on the mother–infant physiological and behavioral attunement.

Moreover, previous research reporting the effectiveness of mindfulness-based interventions in improving the quality of the mother–infant relationship used mainly maternal-reports (e.g., Potharst et al., 2017; Shreffler et al., 2019). Those results, with our reported reduction in parenting stress, might be interpreted as an increased awareness fostered by mindfulness practice that leads mothers to be more aware of their parenting abilities, reporting a more accurate evaluation of the quality of the relationship with their own infants. More empirical findings are needed to confirm whether this increased awareness also corresponds to a better-observed quality of the interaction.

Furthermore, it is possible that the effect of the intervention is more evident during a stressful condition, a situation in which the effect of mindfulness may be stronger, than during face-to-face interactions as the condition explored in the present study. Future studies should take this into account.

Finally, mothers who attended the mindfulness-based intervention reported only a slight improvement in their dispositional mindfulness. The mindfulness literature underlined the limit of mindfulness self-reports that are often biased with mothers more aware of their present-moment internal experience also more aware of the moments in which they are not and often reporting lower mindfulness. This could contribute to the stability of the scores of mindful parenting (Per et al., 2020). Additionally, it must be considered that our sample consisted of low-risk mothers with high levels of mindfulness at baseline. Future observational research should investigate the effect of mindfulness-based interventions in clinical at-risk samples (e.g., depressed or anxious mothers).

The current exploratory findings should be interpreted in the context of several limitations. First, in both studies, our sample was relatively small and only composed by self-selected mothers limiting the generalizability of findings to fathers and to other mothers less interested in the topic. The effects suggested here should be explored in larger samples. Moreover, we assessed the self-reported number of audios listened by mothers, which makes it difficult to know the trustworthiness of this information. This aspect may have adversely influenced our findings. Finally, we explored the effects of mindfulness only on face-to-face free-play interactions. As evidenced by previous work (Laurent et al., 2017), the positive effect of maternal mindfulness could be stronger when mothers have to deal with a stressed infant, e.g., during the still-face paradigm. Additionally, at 9 months of age the effect of mindfulness could be more pronounced when considering other interactive dimensions (i.e., triadic interaction) or exploring the effects on infant socio-emotional development. Future longitudinal studies should explore the effects of mindful parenting and maternal dispositional mindfulness on the quality of mother–infant physiological and behavioral attunement comparing face-to-face, play with toys interactions, and stressful interactive conditions.

Despite these limits, our exploratory research has several strengths. Research studying the role of maternal mindfulness on observed mother–infant interaction is yet scarce. While previous studies have mainly focused on mindful parenting, large age samples, and on mother–infant physiological and behavioral functioning separately, this is the first study that highlights the protective role of mindfulness on both levels of mother–infant interaction simultaneously with observational measures at 4 and 9 months of infant age.

Even if our exploratory studies are composed by small and self-selected mothers’ samples, both suggested that maternal dispositional mindfulness and mindful parenting are important protective factor that could support mothers to better attune to infant physiological and behavioral cues, a crucial maternal ability to promote a positive mother–infant interaction and to support infant emotional development (Stern et al., 1985; Feldman, 2007). Thus, mindfulness could be applied in screenings aimed to identify more at-risk dyads. Available interventions developed with the aim to promote positive parenting (Juffer et al., 2018) should consider maternal mindfulness as an individual factor that could affect the ability of the parent to attune to infants’ signals and therefore to be sensitive to their needs. Furthermore, mindfulness-based interventions could be offered mainly to more at-risk mothers in order support them in managing stress, enhancing emotion regulation and wellbeing, and in developing more appropriate dyadic interactions with their own infants. Indeed, these interventions could equip parents with effective coping strategies and practical tools that could be integrated into daily routines to deal with the challenges of parenting (e.g., deep breathing to stay calm during stressful moments and/or fully engaging in activities with the child). Because interventions based on mindfulness practices, especially those as our conducted with audios, are cheap and simple to implement, they may be particularly suitable for mothers of young infants who are already overburdened and have little time.

Finally, considering that our sample included non-clinical mothers, future studies investigating the effect of mindfulness-based interventions in at-risk populations could contribute to interesting insights for applied settings. These interventions may be particularly effective for at-risk populations and may significantly prevent the effects of maternal psychological difficulties (e.g., depression or anxiety) on mother–infant attunement, with cascade consequences in socio-emotional infant developmental domains.

## Data availability statement

The raw data supporting the conclusions of this article will be made available by the authors, without undue reservation.

## Ethics statement

The studies involving humans were approved by Department of Neuroscience, Imaging and Clinical Sciences, University G. d’Annunzio Chieti-Pescara. The studies were conducted in accordance with the local legislation and institutional requirements. Written informed consent for participation in this study was provided by the participants’ legal guardians/next of kin.

## Author contributions

IP: Data curation, Methodology, Writing – original draft. MP: Formal analysis, Methodology, Writing – review & editing. ON: Writing – review & editing. FL: Writing – review & editing. GD’U: Writing – review & editing. RP: Writing – review & editing. MF: Writing – review & editing. MS: Conceptualization, Methodology, Supervision, Writing – original draft.
