# Supplementary material for: From inner to dyadic connection: the role of mindfulness in mother–infant interaction during the first year of life
Source: Front Behav Neurosci. 2024 Aug 8;18:1398042. doi: 10.3389/fnbeh.2024.1398042 (PMC11338867; doi:10.3389/fnbeh.2024.1398042)

**Supplementary**

**S.1. Study 1 Priors Speciﬁcation**

For both types of models (linear and beta), we utilized non-informative priors. More speciﬁcally, the priors for regression coeﬃcients in linear models were set as Normal (0, 2.5), and for residual standard deviations, Exponential (1) was used. In beta models, the priors for regression coeﬃcients were set as in linear models, Normal (0, 2.5), for residual standard deviations were set as truncated Student’s t (3, 0, 2.5) and the priors for the φ parameters were set as Gamma (0.01, 0.01).

**S.2. Study 1 Models Analysis Plan**

For the analyses we proceeded with the following steps:

1. Fitting models. We ﬁtted all models using the speciﬁed priors. For MCMC sampling we adopted 4 chains of 8000 replicates each, with 4000 as warmup. Consequently, the eﬀective number of posterior samples was 16000. MCMC convergences were assessed by means of the Potential Scale Reduction Statistic (PSRF or Rhat; Gelman & Rubin, 1992).
2. Diagnostics. In addition to the traditional residuals diagnostics plots (for linearity, normality and ho­moscedasticity), we adopted the Posterior Predictive Check (PPC), this latter is a graphical comparison between data simulated from the posterior predictive distribution and real-world observations; if a model is a good ﬁt then generated data looks a lot like the observed data (Gabry, Simpson, Vehtari, Betancourt, & Gelman, 2019).
3. Model comparison. We used the following indices: Leave-one-out cross validation criterion (LOO; Vehtari, Gelman, & Gabry, 2017), model weights (W; Yao, Vehtari, Simpson, & Gelman, 2018) and *R2* – one for each response variable – with 90% credible intervals. Credible intervals are intervals containing a speciﬁc portion of posterior distribution; in our case, they represent the set of more plausible values for *R2*, with a chosen probability. As all Information Criteria, LOO estimates the model predictive capability; the lower the value, the better will be the model. Model weights are values ranging from zero to one that can be interpret as an estimate of the probability that the model will make the best predictions on new data, conditional on the set of models considered (Burnham & Anderson, 2002). Therefore, in the set of considered models, the best will be the one with lowest LOO and highest W.
4. Best Model analysis. Finally, we selected the best model and analysed it in detail. In particular, we analysed the model parameters and predictions based on the parameters posterior distributions.

**S.3. Study 1 Descriptives**

In this section, we report univariate (Table S1) and bivariate (Figure S1) descriptive statistics of the target variables.

**Table S1.** *Univariate Descriptives Statistics of the Target Variables.*

|  | *M* | *SD* | *Median* | *Trimmed* | *Mad* | *Min* | *Max* | *Range* | *Skew* | *Kurtosis* | *se* |
| --- | --- | --- | --- | --- | --- | --- | --- | --- | --- | --- | --- |
| Mother RSA | 7.43 | 2.06 | 8.07 | 7.59 | 2.28 | 2.73 | 9.99 | 7.26 | -0.49 | -1.10 | 0.11 |
| Symmetrial Co-regulation | 0.28 | 0.33 | 0.15 | 0.23 | 0.22 | 0.00 | 1.00 | 1.00 | 0.90 | -0.50 | 0.02 |
| Unilateral Co-regulation | 0.29 | 0.37 | 0.00 | 0.24 | 0.00 | 0.00 | 1.00 | 1.00 | 0.86 | -0.81 | 0.02 |
| Infant RSA | 6.77 | 2.48 | 7.46 | 6.94 | 2.82 | 1.91 | 9.85 | 7.93 | -0.43 | -1.28 | 0.14 |
| IMP Total Score | 3.79 | 0.36 | 3.73 | 3.78 | 0.36 | 3.07 | 4.52 | 1.45 | 0.22 | -0.66 | 0.02 |
| FFMQ Total Score | 3.59 | 0.45 | 3.49 | 3.58 | 0.44 | 2.28 | 4.56 | 2.28 | -0.05 | 0.23 | 0.02 |

**Figure S1.** *Pairs Plot of Target Variables.*


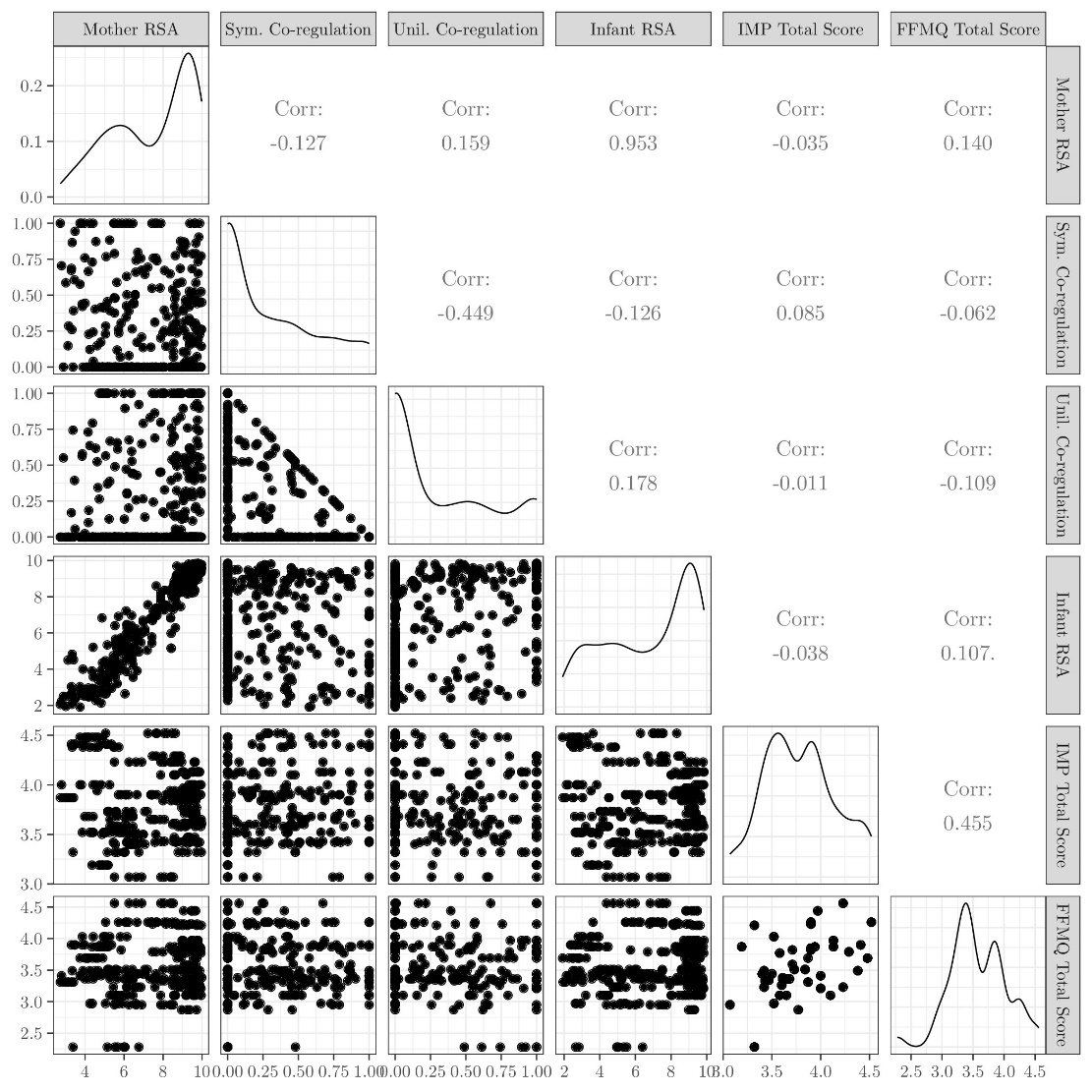


*Note.* On the main diagonal, univariate densities are depicted. On the other graphs, below the diagonal, pairwise bivariate scatterplots are shown. Above the diagonal, correlation coefficients are reported.

**S.4. Study 1 Models' Diagnostics: Outcome Mother RSA**

Table S1 presents the comparisons for models with Mother RSA as outcome.

Figures S2 and S3 represent the diagnostic plots for the two models discussed in section 2.2.2.

The four panels represent: 1) Residuals vs Fitted values, where in a good model, reference lines should be at and horizontal; 2) Normal Q-Q plot, where in a good model, dots should fall along the line; 3) Scale-Location plot, where reference lines should be at and horizontal; 4) Posterior Predictive Check, where model predicted densities should resemble the observed density line.

|  | Model | LOO | SE | W | R^2^ |
| --- | --- | --- | --- | --- | --- |
| M09a | Infant RSA x IMP Total Score + (1\|ID) | 600.26 | 42.57 | 0.23 | 0.92 |
| M10a | Infant RSA x FFMQ Total Score + (1\|ID) | 600.35 | 43.26 | 0.22 | 0.92 |
| M06a | Infant RSA + FFMQ Total Score + (1\|ID) | 600.99 | 43.37 | 0.15 | 0.92 |
| M01a | Infant RSA + (1\|ID) | 601.53 | 43.02 | 0.12 | 0.92 |
| M05a | Infant RSA +IMP Total Score + (1\|ID) | 601.87 | 42.94 | 0.10 | 0.92 |
| M11a | Infant RSA x IMP Total Score + Time + (1\|ID) | 602.73 | 42.57 | 0.07 | 0.92 |
| M12a | Infant RSA x FFMQ Total Score + Time + (1\|ID) | 602.87 | 43.252 | 0.06 | 0.92 |
| M04a | Infant RSA + Time + (1\|ID) | 603.55 | 43.06 | 0.044 | 0.92 |
| M07a | FFMQ Total Score + Time + (1\|ID) | 761.15 | 45.56 | 2.64 | 0.88 |
| M08a | IMP Total Score + Time + (1\|ID) | 761.20 | 45.39 | 2.57 | 0.88 |
| M02a | IMP Total Score + (1\|ID) | 772.48 | 48.06 | 9.12 | 0.87 |
| M00a | (1\|ID) | 772.62 | 48.10 | 8.52 | 0.87 |
| M03a | FFMQ Total Score + (1\|ID) | 772.78 | 47.68 | 7.86 | 0.87 |

**Table S2.** *Model Comparison for Mother RSA.*

*Note.* Each row provides information on the model formula, LOO (with standard errors), model weights (W), and R^2^. ID = subject ID code.

**Figure S2.** *Model M09a Diagnostics Plots.*


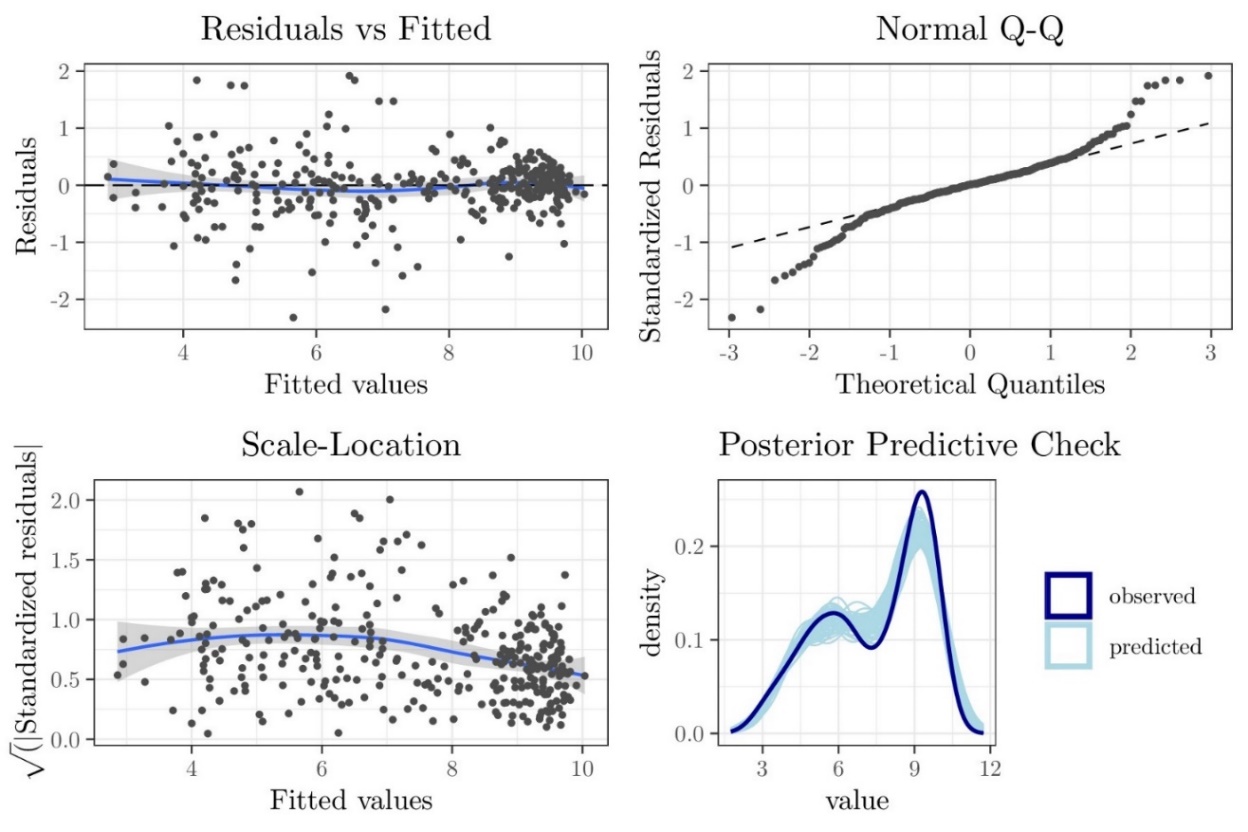


**Figure S3.** *Model M10a Diagnostics Plots.*


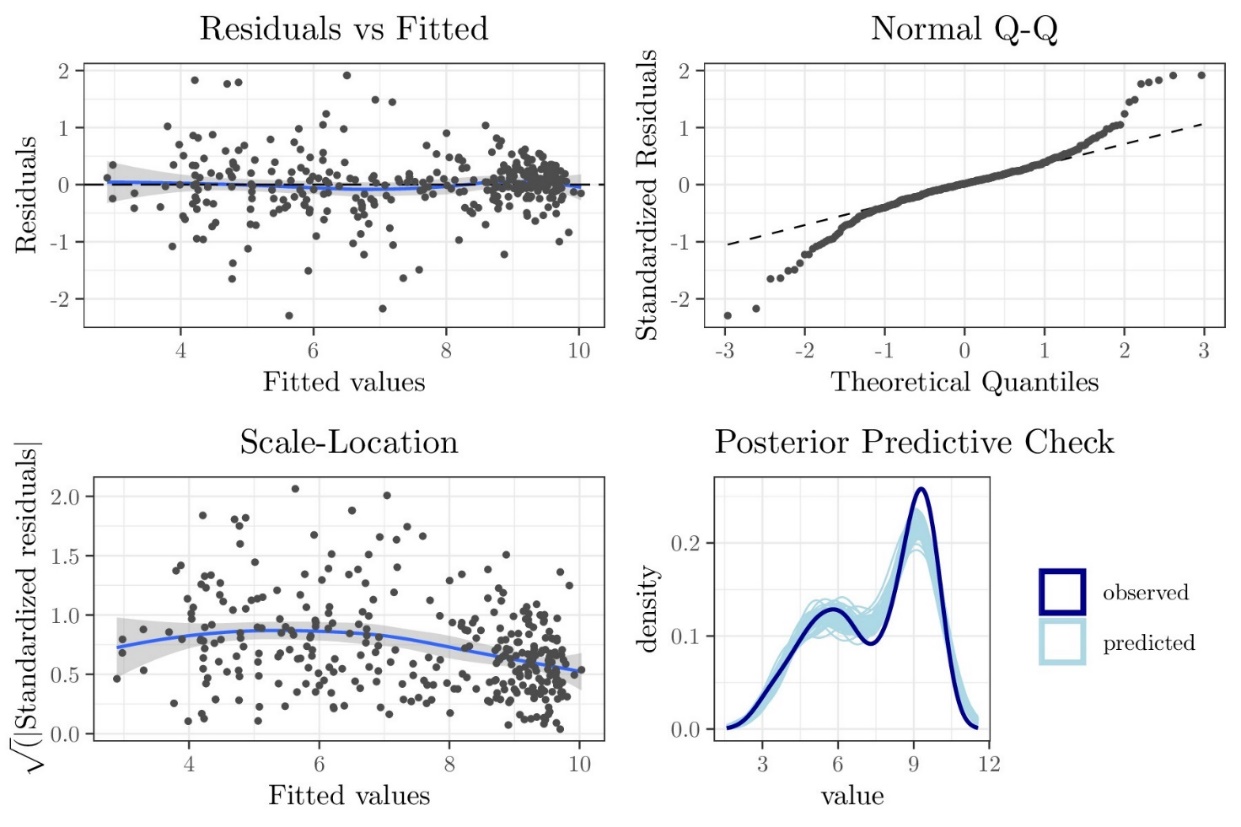


**S.5. Study 1 Models' Diagnostics: Outcome Symmetrical and Unilateral Co-regulation**

Table S3 presents the comparisons for models with Mother RSA as outcome.

Figures S4, S5, S6, and S7 represent the diagnostic plots for the two models discussed in section 2.2.3.

The four panels represent: 1) Residuals vs Fitted values, where in a good model, reference lines should be at and horizontal; 2) Normal Q-Q plot, where in a good model, dots should fall along the line; 3) Scale-Location plot, where reference lines should be at and horizontal; 4) Posterior Predictive Check, where model predicted densities should resemble the observed density line.

**Table S3.** *Model Comparison for Symmetrical and Unilateral Co-regulation.*

|  | Model | LOO | SE | W | Symmetrical | Unilateral |
| --- | --- | --- | --- | --- | --- | --- |
| M08b | IMP Total Score + Time + (1\|ID) | -13027.30 | 510.44 | 0.34 | 0.30 | 0.36 |
| M07b | FFMQ Total Score + Time + (1\|ID) | -13026.48 | 510.40 | 0.22 | 0.30 | 0.36 |
| M14b | FFMQ Total Score x Infant RSA + Time + (1\|ID) | -13025.64 | 510.49 | 0.15 | 0.30 | 0.36 |
| M06b | Mother RSA + Time + (1\|ID) | -13024.46 | 510.52 | 0.08 | 0.30 | 0.37 |
| M05b | Infant RSA + Time + (1\|ID) | -13023.48 | 510.57 | 0.05 | 0.30 | 0.37 |
| M18b | Mother RSA x Infant RSA + Time + (1\|ID) | -13022.60 | 510.45 | 0.03 | 0.32 | 0.36 |
| M16b | FFMQ Total Score x Mother RSA + Time + (1\|ID) | -13022.46 | 510.31 | 0.03 | 0.32 | 0.36 |
| M15b | IMP Total Score x Infant RSA + Time + (1\|ID) | -13021.46 | 510.56 | 0.02 | 0.32 | 0.37 |
| M00b | (1\|ID) | -13021.21 | 510.62 | 0.02 | 0.33 | 0.37 |
| M03b | FFMQ Total Score + (1\|ID) | -13020.99 | 510.63 | 0.01 | 0.30 | 0.38 |
| M04b | IMP Total Score + (1\|ID) | -13020.70 | 510.60 | 0.01 | 0.30 | 0.37 |
| M17b | IMP Total Score x Mother RSA + Time + (1\|ID) | -13020.57 | 510.53 | 0.01 | 0.31 | 0.37 |
| M09b | FFMQ Total Score x Infant RSA + (1\|ID) | -13019.63 | 510.68 | 0.01 | 0.31 | 0.37 |
| M02b | Mother RSA + (1\|ID) | -13019.23 | 510.70 | 0.01 | 0.31 | 0.37 |
| M01b | Infant RSA + (1\|ID) | -13018.65 | 510.80 | 0.00 | 0.33 | 0.38 |
| M11b | FFMQ Total Score x Mother RSA + (1\|ID) | -13017.14 | 510.47 | 0.00 | 0.33 | 0.37 |
| M13b | Mother RSA x Infant RSA + (1\|ID) | -13017.06 | 510.58 | 0.00 | 0.33 | 0.37 |
| M10b | IMP Total Score x Infant RSA + (1\|ID) | -13015.00 | 510.72 | 0.00 | 0.33 | 0.37 |
| M12b | IMP Total Score x Mother RSA + (1\|ID) | -13014.67 | 510.75 | 0.00 | 0.34 | 0.36 |

*Note.* Each row provides information on the model formula, LOO (with standard errors), model weights (W), and R2, one for each dependent variable. ID = subject ID code.

**Figure S4.** *Model M08b Diagnostics Plots (for Symmetrical Co-regulation).*


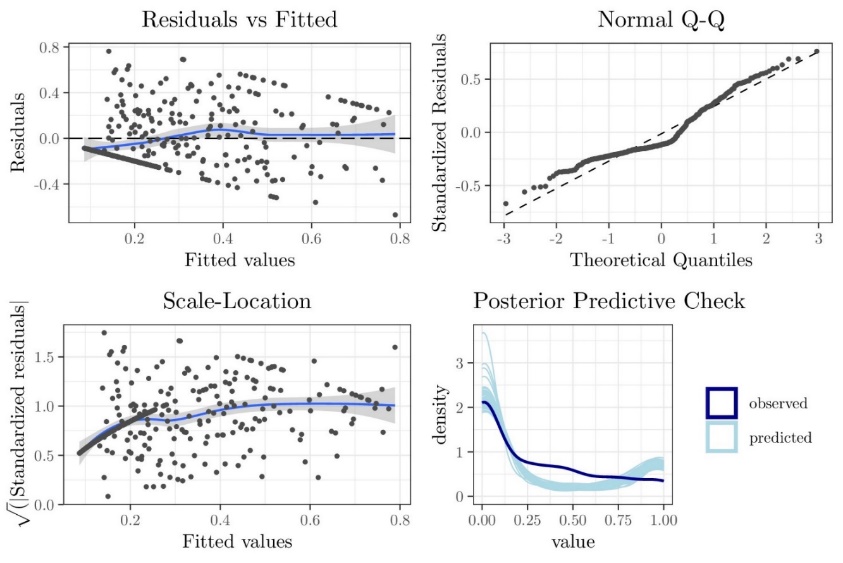


**Figure S5.** *Model M08b Diagnostics Plots (for Unilateral Co-regulation).*


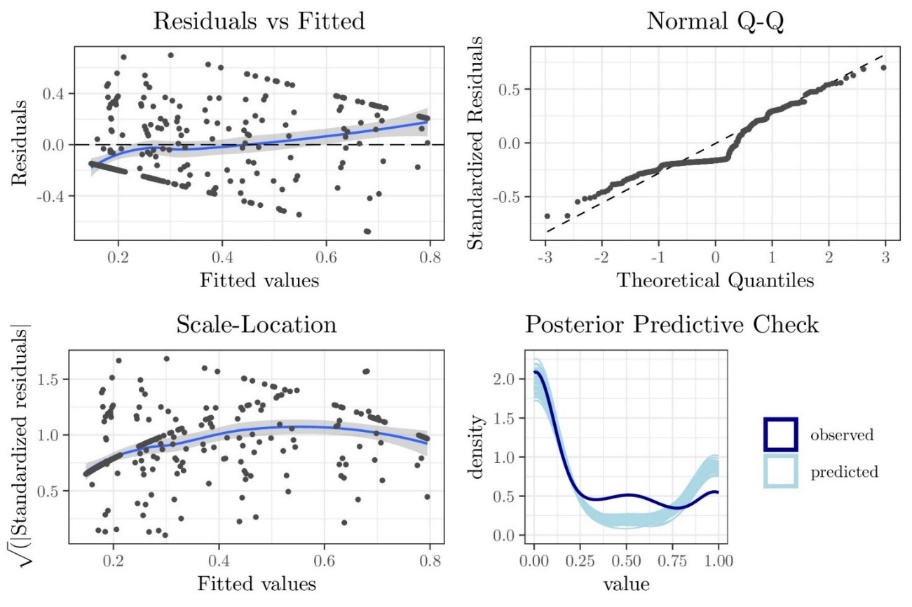


**Figure S6.** *Model M07b Diagnostics Plots (for Symmetrical Co-regulation).*


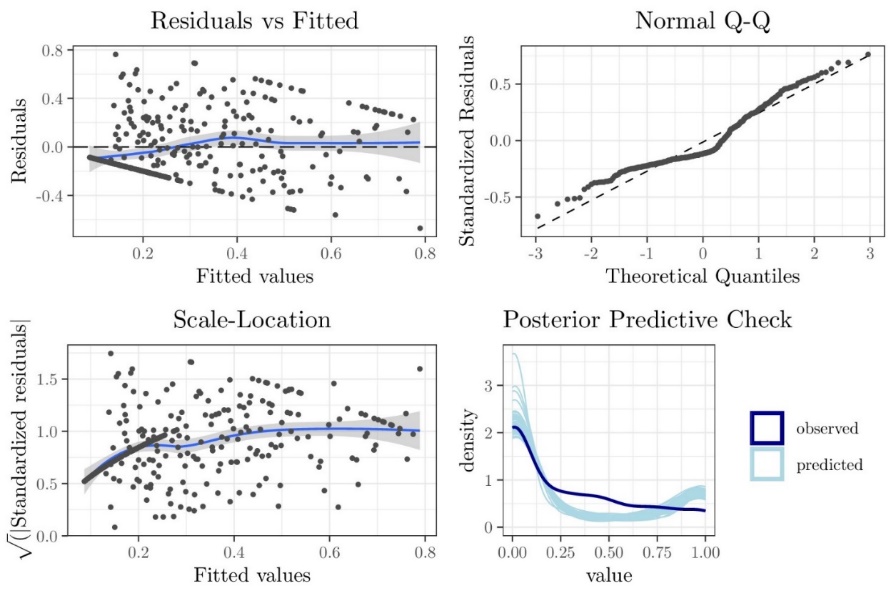


**Figure S7.** *Model M07b Diagnostics Plots (for Unilateral Co-regulation).*


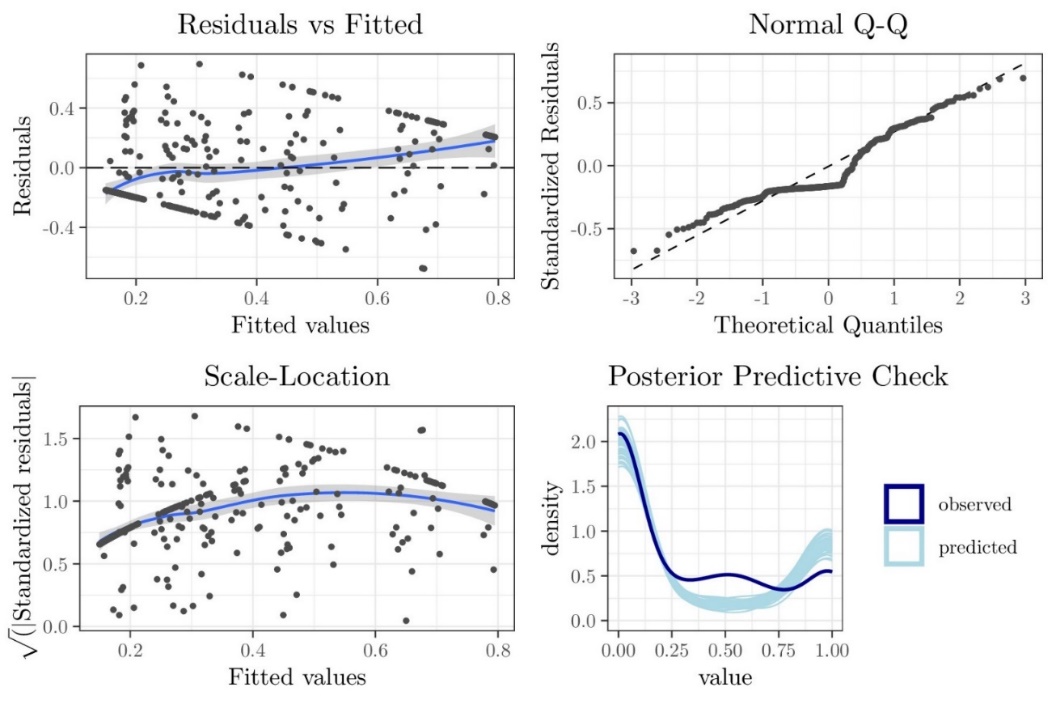


**S.6. Study 2 Univariate Statistics**

Table S4 shows univariate statistics (means, standard deviations, and correlations) among study 2 variables at T1. Statistics are computed on 55 dyads (37 dyads for RSA correlations).

**Table S4.** *Univariate statistics of mindfulness, parenting stress, and mother-infant interaction variables at T1 (N = 55).*

|  |  | *M* | *SD* | 1 | 2 | 3 | 4 | 5 | 6 | 7 | 8 | 9 | 10 | 11 |
| --- | --- | --- | --- | --- | --- | --- | --- | --- | --- | --- | --- | --- | --- | --- |
| 1 | RSA corr. | 0.50 | 0.49 | — |  |  |  |  |  |  |  |  |  |  |
| 2 | Mother Positive | 0.44 | 0.18 | .077 | — |  |  |  |  |  |  |  |  |  |
| 3 | Mother Positive Voc. | 0.40 | 0.12 | -.033 | -.747 | — |  |  |  |  |  |  |  |  |
| 4 | M. Positive-I. Positive | 0.10 | 0.08 | -.102 | .605 | -.503 | — |  |  |  |  |  |  |  |
| 5 | M. Positive Voc-I. Positive | 0.02 | 0.03 | -.139 | .111 | -.065 | .518 | — |  |  |  |  |  |  |
| 6 | Attentive Match | 0.31 | 0.18 | .084 | .447 | -.247 | .634 | .420 | — |  |  |  |  |  |
| 7 | Affective Match | 0.48 | 0.16 | .138 | -.358 | .326 | .097 | .206 | .239 | — |  |  |  |  |
| 8 | IMP Total Score | 3.75 | 0.40 | .177 | -.100 | .210 | -.063 | .173 | .075 | .035 | — |  |  |  |
| 9 | FFMQ Total Score | 3.45 | 0.47 | .156 | -.071 | .287 | -.167 | -.021 | -.006 | -.013 | .672 | — |  |  |
| 10 | PSI PD | 2.19 | 0.62 | -.047 | -.028 | -.033 | -.148 | -.279 | -.253 | -.261 | -.426 | -.398 | — |  |

*Note.* RSA corr: RSA correlation; Mother Positive Voc: Mother Positive Vocalization; M. Positive-I. Positive: Mother Positive-Infant Positive; M. Positive Voc-I. Positive: Mother Positive Vocalization-Infant Positive; IMP Total Score: Interpersonal Mindfulness in Parenting Total Score; FFMQ Total Score: Five Facet Mindfulness Questionnaire Total Score; PSI PD: Parenting Stress Index Parental Distress.

|  |
| --- |
|  |

**Table S5.** *Univariate Descriptives Statistics of the Target Variables*

|  | | *n* | *M* | *SD* | *Median* | *Trimmed* | *Mad* | *Min* | *Max* | *Range* | *Skew* | *Kurtosis* | *se* |
| --- | --- | --- | --- | --- | --- | --- | --- | --- | --- | --- | --- | --- | --- |
| IMP Total Score | 101.00 | | 3.76 | 0.40 | 3.77 | 3.76 | 0.43 | 2.94 | 4.65 | 1.71 | 0.01 | - 0.61 | 0.04 |
| FFMQ Total Score | 99.00 | | 3.48 | 0.49 | 3.41 | 3.47 | 0.49 | 2.38 | 4.68 | 2.30 | 0.26 | - 0.19 | 0.05 |
| PSI PD | 105.00 | | 2.18 | 0.66 | 2.17 | 2.14 | 0.62 | 1.00 | 4.00 | 3.00 | 0.51 | - 0.01 | 0.06 |
| RSA corr. | 78.00 | | 0.54 | 0.46 | 0.69 | 0.62 | 0.31 | - 1.00 | 0.98 | 1.98 | - 1.62 | 2.23 | 0.05 |
| Mother Positive | 104.00 | | 0.44 | 0.18 | 0.45 | 0.44 | 0.21 | 0.09 | 0.89 | 0.80 | 0.18 | - 0.73 | 0.02 |
| Mother Positive Voc. | 104.00 | | 0.41 | 0.13 | 0.41 | 0.42 | 0.14 | 0.08 | 0.69 | 0.61 | - 0.18 | - 0.67 | 0.01 |
| M. Positive-I. Positive | 103.00 | | 0.10 | 0.08 | 0.09 | 0.09 | 0.08 | 0.00 | 0.38 | 0.38 | 1.03 | 0.99 | 0.01 |
| M. Positive Voc-I. Positive | 103.00 | | 0.02 | 0.02 | 0.01 | 0.01 | 0.01 | 0.00 | 0.16 | 0.16 | 3.08 | 13.71 | 0.00 |
| Attentive Match | 103.00 | | 0.33 | 0.18 | 0.29 | 0.31 | 0.16 | 0.03 | 0.81 | 0.78 | 0.61 | - 0.29 | 0.02 |
| Affective Match | 103.00 | | 0.47 | 0.16 | 0.47 | 0.48 | 0.17 | 0.10 | 0.82 | 0.71 | - 0.14 | - 0.32 | 0.02 |

**S6. Study 2 Priors Specification**

For interaction parameters, we adopted a Student's t distribution, specifically t(3, 0.15, 0.41) (Figure S8(A)) for mindfulness and parenting stress variables and t(3, 0.05, 0.198) (Figure S8(B)) for mother-infant interaction variables, where the parameters represent degrees of freedom, location, and scale, respectively. We choose these distributions to define specific prior probabilities for two target intervals of interaction parameters: specifically, *P*(*β* Є [-0.05; 0.3]) ≈ 0.3 for mindfulness and parenting stress variables (IMP Total Score, FFMQ Total Score, and PSI PD) and *P*(*β* Є [-0.01; 0.1]) ≈ 0.2 for mother-infant interaction variables, where *β* represents the interaction parameter. The target intervals, [-0.05; 0.3] and [-0.01; 0.1, respectively, were selected under the hypothesis that expected interactions would be small. For the other regression coefficients, we employed a weakly informative prior, specifically a Student's t(3, 0, 1) distribution (Figure S8[C]).

**S.6. Study 2 Outcome: Mindfulness and Parenting Stress Variables**

**Table S6.** *Outcome Mindfulness and Parenting Stress Variables: Interaction (Intervention Condition X Session) Parameter Estimates with 90% Credibility Interval (CI).*

| Model | *Estimate* | *95% C.I.* | *R^2^* |
| --- | --- | --- | --- |
| IMP Total Score | 0.01 | -0.12 - 0.14 | 0.78 |
| FFMQ Total Score | 0.11 | -0.06 - 0.29 | 0.73 |
| PSI PD | -0.14 | -0.37 - 0.1 | 0.69 |

**S.7. Study 2 Outcome: Mother-Infant Interaction Variables**

**Table S7.** *Outcome Behavioral Variables: Interaction (Intervention Condition X Session) Parameter Estimates with 90% Credibility Interval (CI).*

| Model | *Estimate* | *95% C.I* | *R^2^* |
| --- | --- | --- | --- |
| RSA corr. | 0.08 | -0.164 - 0.323 | 0.10 |
| Mother Positive | -0.04 | -0.126 - 0.042 | 0.51 |
| Mother Positive Voc. | 0.03 | -0.32 - 0.102 | 0.43 |
| M. Positive-I. Positive | -0-02 | -0.061 - 0.014 | 0.53 |
| M Positive Voc-I. Positive | 0.00 | -0.013 - 0.016 | 0.12 |
| Attentive Match | -0.01 | -0.084 - 0.063 | 0.61 |
| Affective Match | 0.08 | -0-01 - 0.159 | 0.33 |

**Figure S8.** *Priors Distributions for Regression Parameters.*


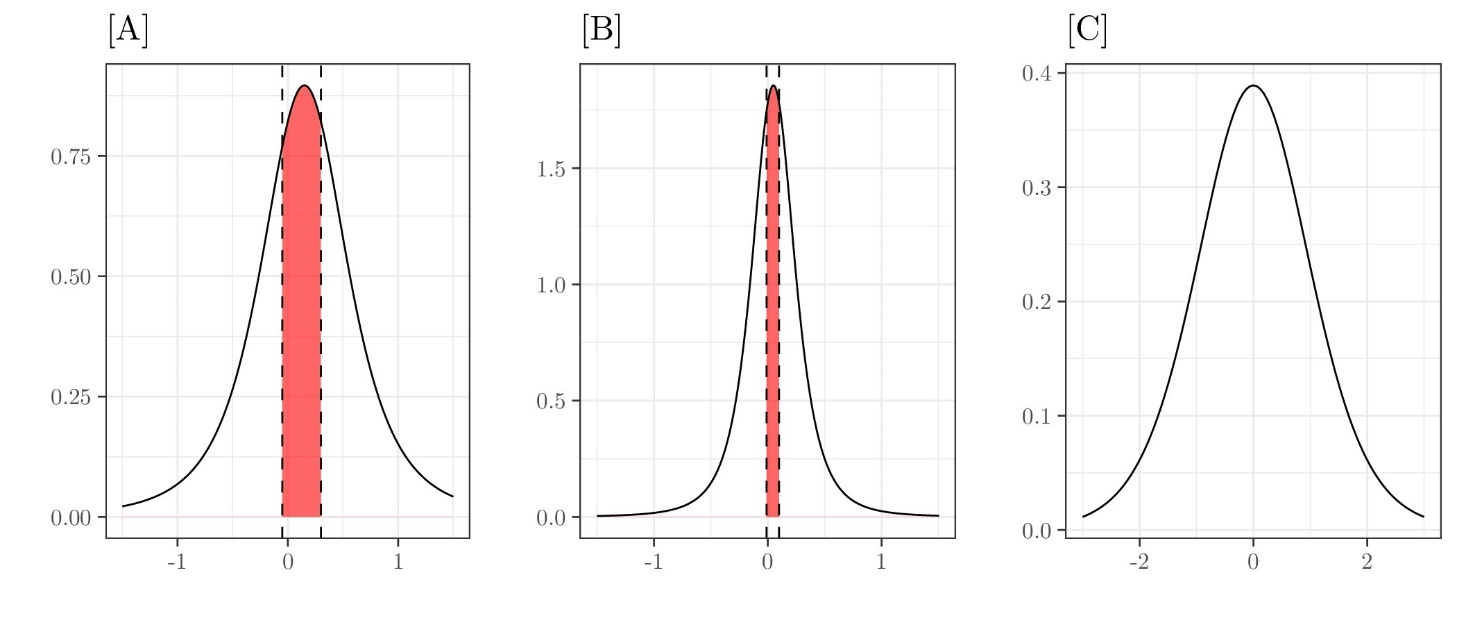


*Note.* [A] Prior of interaction parameter for mindfulness variables, [B] Prior of interaction parameter for behavioural variables, and [C] Prior for other regression parameters. The red area in panels [A] and [B] represents the prior probability assigned to the target intervals for the interaction parameter, namely 0.3 and 0.2, respectively.

**Figure S9.** *Mindfulness and Parenting Stress Variables. Expected Values (with 90% HDI) of the Models as a Function of Session (in Abscissa) and Intervention condition (Colors).*


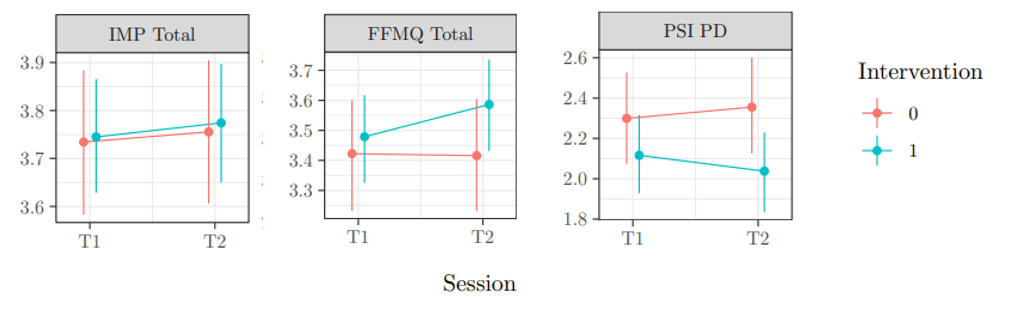


*Note.* IMP Total: IMP Total Score; FFMQ Total: FFMQ Total Score; PSI PD: PSI Parental Distress

**Figure S10.** *Mindfulness Variables. Posterior of Interactions (Black Lines) compared with Prior (Red Lines).*


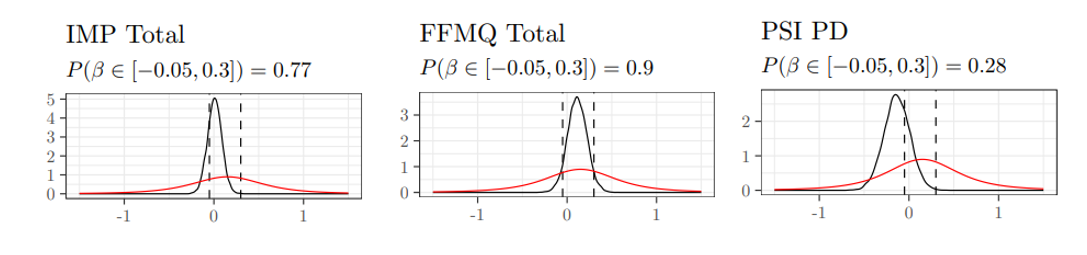


**Figure S11.** *IMP Total Score Model Diagnostics.*


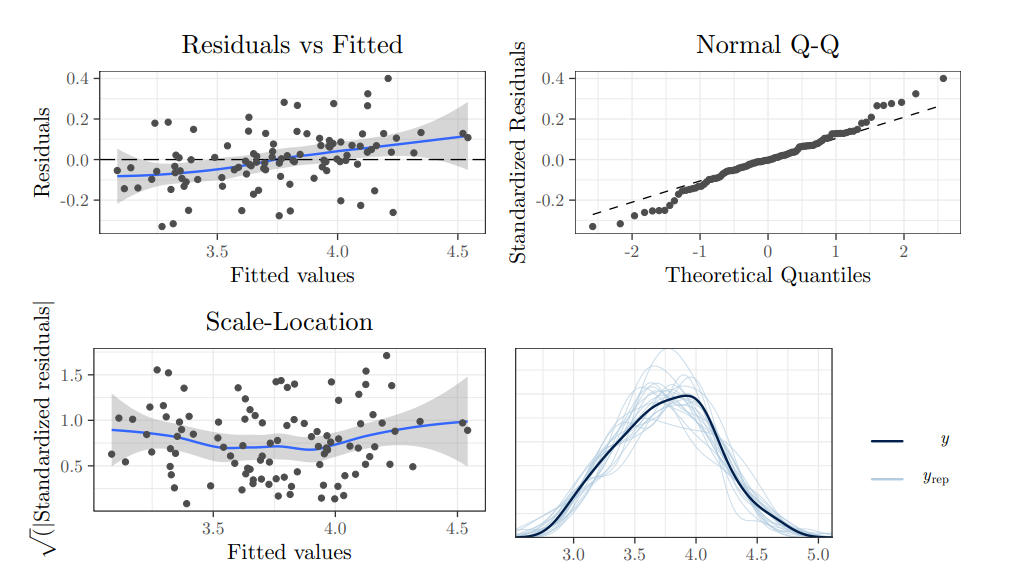


**Figure S12.** *FFMQ Total Score Model Diagnostics.*


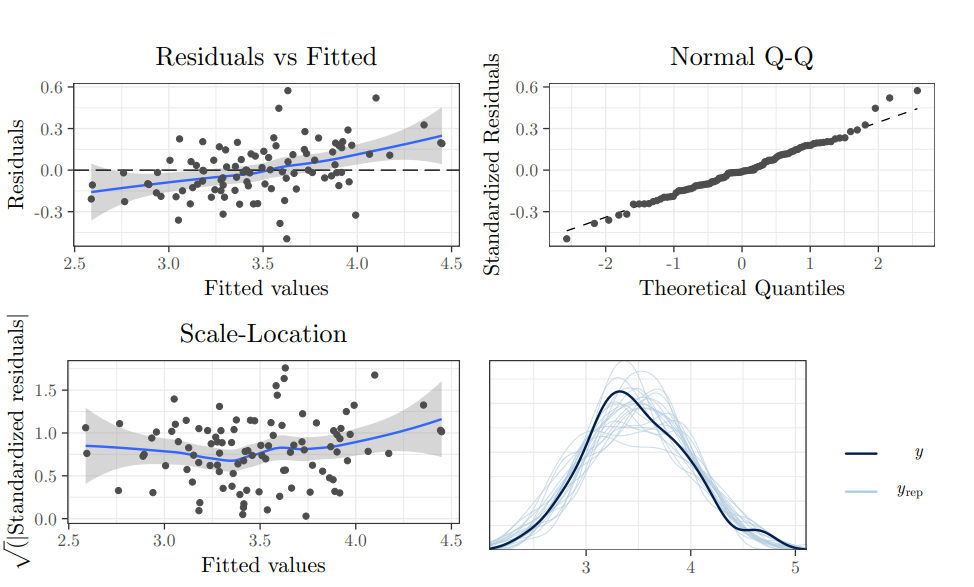


**Figure S13.** *PSI PD Model Diagnostics.*


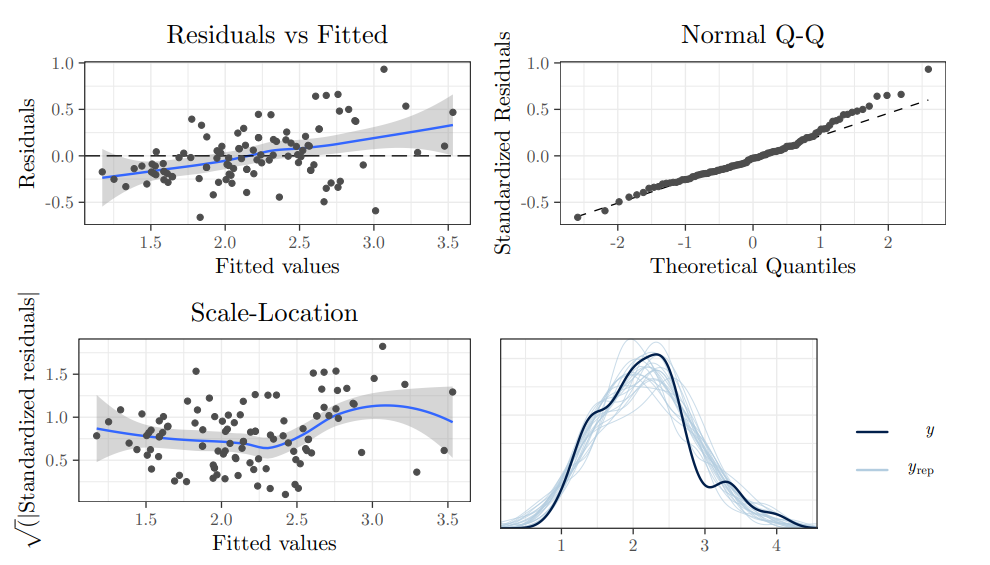


**Fig. S14.** *Behavioural Variables.* *Expected Values (with 90% HDI) of the Models as a Function of Session (in Abscissa) and Intervention Condition (Colors).*


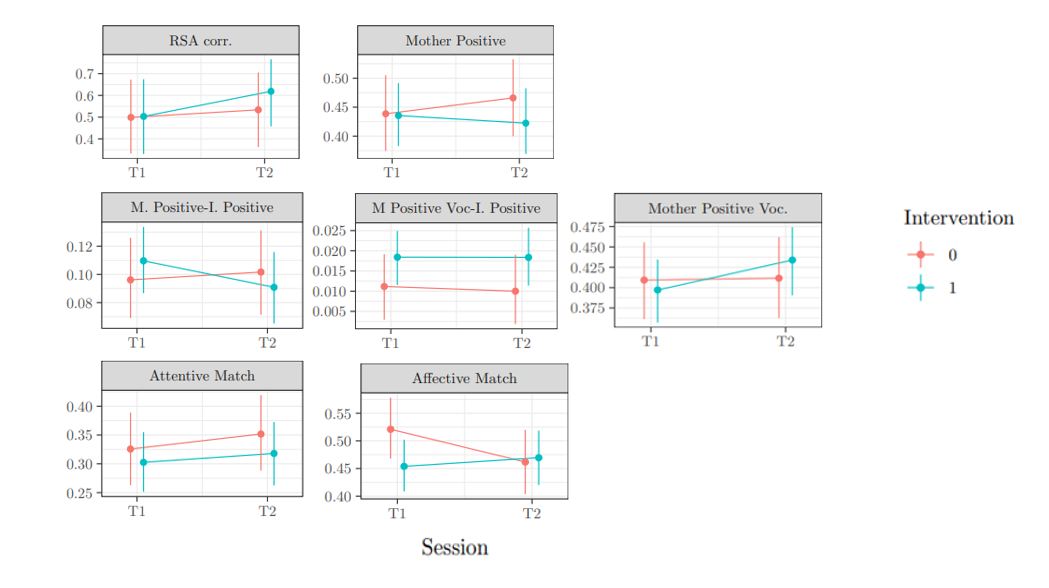


**Fig. S15.** *Behavioural Variables. Posterior of Interactions (Black Lines) compared with Prior (Red Lines).*


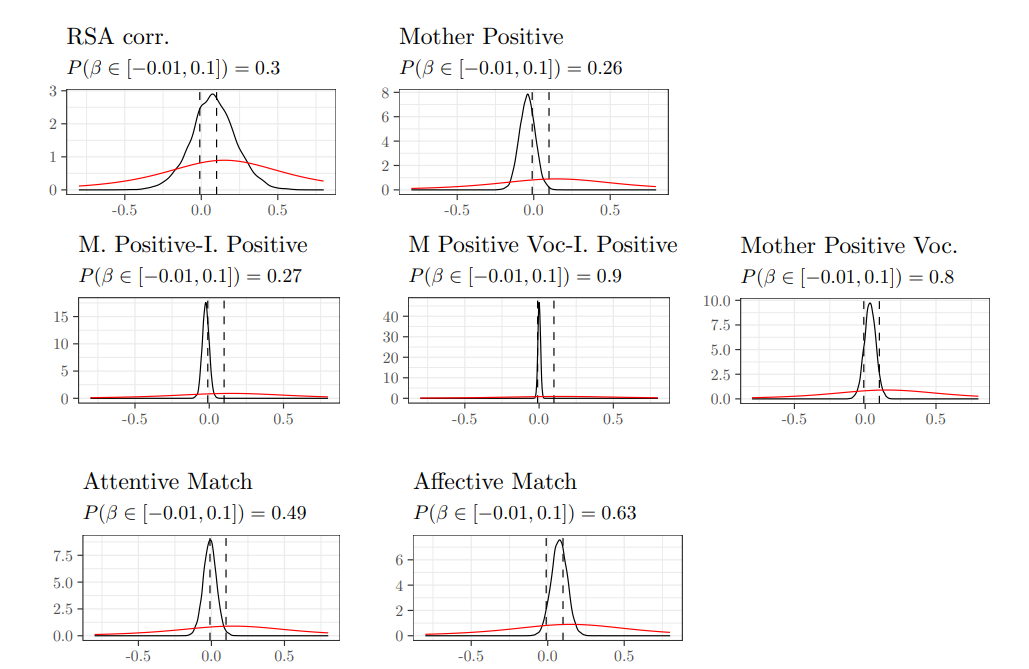


**Figure S16.** *RSA Correlations Model Diagnostics.*


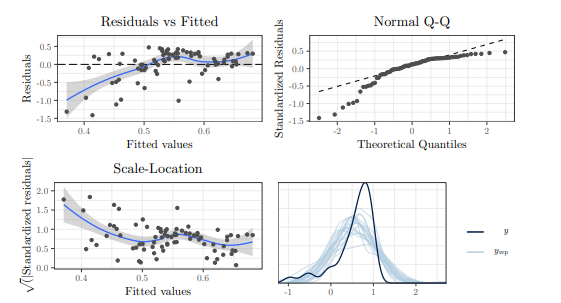


**Figure S17.** *Mother Positive Correlations Model Diagnostics.*


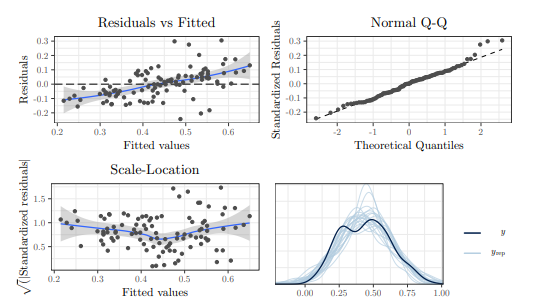


**Figure S18.** *Mother Positive Voc. Correlations Model Diagnostics.*


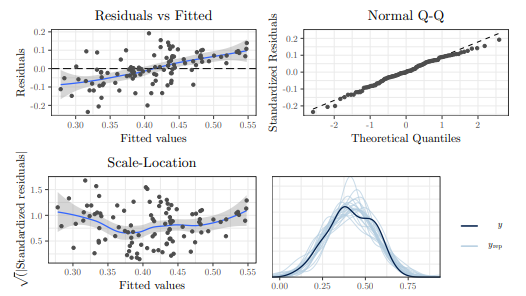


**Figure S19.** *M. Positive-I. Positive Correlations Model Diagnostics.*


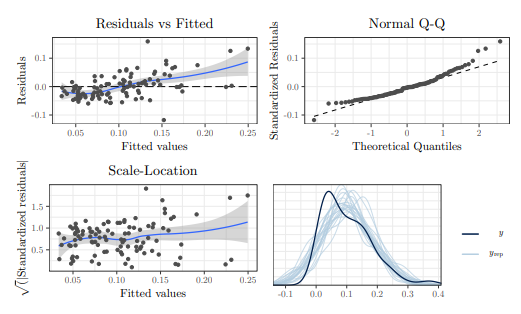


**Figure S20.** *M. Positive Voc.-I. Positive Correlations Model Diagnostics.*


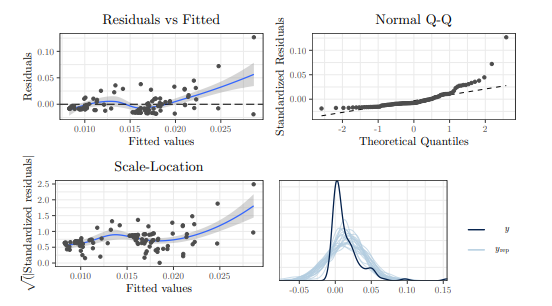


**Figure S21.** *Attentive Match Correlations Model Diagnostics.*

*
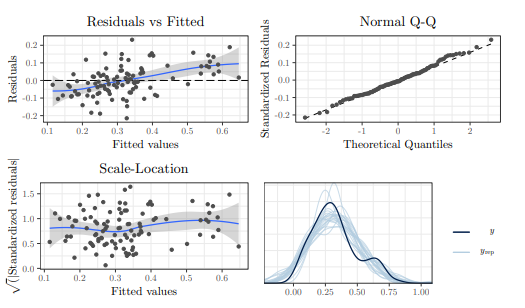
*

**Figure S22.** *Affective Match Correlations Model Diagnostics.*


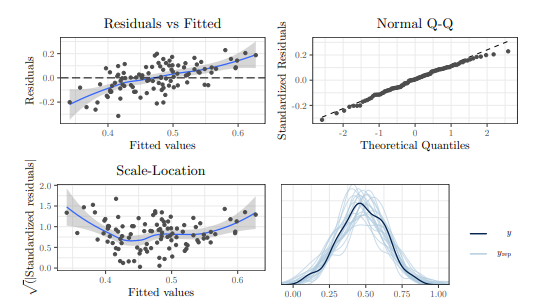

Supplement: Supplementary file 1 [file Data_Sheet_1.docx]
